# CTRP3 alleviates mitochondrial dysfunction and oxidative stress injury in pathological cardiac hypertrophy by activating UPRmt via the SIRT1/ATF5 axis

**DOI:** 10.1038/s41420-024-01813-x

**Published:** 2024-01-26

**Authors:** Lei Shi, Yanzhen Tan, Wenying Zheng, Guojie Cao, Haitao Zhou, Panpan Li, Jun Cui, Yujie Song, Lele Feng, Hong Li, Wenju Shan, Bing Zhang, Wei Yi

**Affiliations:** 1 Department of Cardiovascular Surgery, Xijing Hospital, Fourth Military Medical University, Xi’an, 710032 Shaanxi China; 2 Department of General Medicine, Xijing Hospital, Fourth Military Medical University, Xi’an, 710032 Shaanxi China

**Keywords:** Cardiac hypertrophy, Mechanisms of disease

## Abstract

Pathological cardiac hypertrophy is an independent risk factor for heart failure. Disruption of mitochondrial protein homeostasis plays a key role in pathological cardiac hypertrophy; however, the mechanism of maintaining mitochondrial homeostasis in pathological cardiac hypertrophy remains unclear. In this study, we investigated the regulatory mechanisms of mitochondrial protein homeostasis in pathological cardiac hypertrophy. Wildtype (WT) mice, knockout mice, and mice transfected with lentivirus overexpressing mouse C1q-tumor necrosis factor-related protein-3 (CTRP3) underwent transverse aortic constriction or sham surgery. After 4 weeks, cardiac function, mitochondrial function, and oxidative stress injury were examined. For mechanistic studies, neonatal rat cardiomyocytes were treated with small interfering RNA or overexpression plasmids for the relevant genes. CTRP3 overexpression attenuated transverse aortic constriction (TAC) induced pathological cardiac hypertrophy, mitochondrial dysfunction, and oxidative stress injury compared to that in WT mice. TAC or Ang II resulted in compensatory activation of UPRmt, but this was not sufficient to counteract pathologic cardiac hypertrophy. CTRP3 overexpression further induced activation of UPRmt during pathologic cardiac hypertrophy and thereby alleviated pathologic cardiac hypertrophy, whereas CTRP3 knockout or knockdown inhibited UPRmt. ATF5 was a key regulatory molecule of UPRmt, as ATF5 knockout prevented the cardioprotective effect of CTRP3 in TAC mice. In vitro, SIRT1 was identified as a possible downstream CTRP3 effector molecule, and SIRT1 knockout blocked the cardioprotective effects of CTRP3. Our results also suggest that ATF5 may be regulated by SIRT1. Our study demonstrates that CTRP3 activates UPRmt via the SIRT1/ATF5 axis under pathological myocardial hypertrophy, thus attenuating mitochondrial dysfunction and oxidative stress injury.

## Introduction

Pathological cardiac hypertrophy is a common manifestation of cardiovascular disease, including hypertension, pulmonary hypertension, myocardial infarction, arrhythmia, and sudden cardiac death [1]. If not effectively treated, pathological cardiac hypertrophy progresses to heart failure. The long-term survival rate of patients with heart failure remains low; therefore, an improved understanding of the mechanisms underlying pathological cardiac hypertrophy is needed. Although research on effective treatments for heart failure is ongoing, such treatments are not widely available [2].

In response to pathological stimuli, cells undergo stress characterized by a disrupted protein-folding environment within the mitochondria, which leads to the accumulation of unfolded, misfolded, and dysfunctional proteins. This increases reactive oxygen species (ROS) levels as well as induces oxidative stress injury, which further disrupts protein integrity and folding processes; resulting in a vicious cycle of mitochondrial damage [3]. Activation of the mitochondrial unfolded protein response (UPRmt) plays a protective role in cell survival by promoting correct protein folding or inducing hydrolysis of misfolded proteins. Therefore, UPRmt regulation is vital for controlling mitochondrial protein levels [4]. UPRmt in mammalian cells can be activated by a multitude of factors, involving various regulatory mechanisms, such as ATF5, a gene homologous to ATFS-1 that participates in the regulation of UPRmt in nematodes, and UPRmt regulated by the HSF1-SSBP1 complex under heat stress conditions [5, 6]. However, strategies to safely and effectively regulate UPRmt in cardiomyocytes are unknown, as the mechanisms involved are poorly understood.

C1q-tumor necrosis factor-related protein-3 (CTRP3) is one of the most studied members of the CTRP superfamily and is a homodimer of lipocalin, which plays an important role in various diseases and biological processes [7, 8]. We previously showed that CTRP3 could partially reduce pathological cardiac hypertrophy by inhibiting the p38/CREB pathway and p38-induced endoplasmic reticulum stress [9]. In addition, CTRP3 was shown to exert anti-inflammatory, anti-apoptotic, and anti-oxidative stress injury effects whilst being a cardioprotective adipokine [10, 11]. Mitochondria occupy 30% of the total space in cardiomyocytes and supply 95% of the adenosine triphosphate (ATP) required by cardiomyocytes, proving that mitochondrial function status substantially impacts cardiac function [12]. However, the protective effect of CTRP3 against mitochondrial damage in pathological cardiac hypertrophy has not been studied in depth.

SIRT1, belonging to the sirtuin (SIRT) protein family of proteins, is a conserved class III histone deacetylase protein. SIRT1 modifies a variety of target proteins, both histone and non-histone, and is involved in the regulation of a wide range of essential cellular functions, including glycolipid metabolism, mitochondrial biogenesis, DNA repair, oxidative stress, apoptosis, autophagy, cell proliferation, and inflammation [13]. SIRT1 plays a major role in systemic physiology or disease because of its functional diversity. Studies have found that SIRT1 exhibits different or even opposite functions in pathological cardiac hypertrophy. Overexpression of SIRT1 inhibited Ang II-induced pathological cardiac hypertrophy by reducing cardiomyocyte apoptosis and promoting autophagy [14, 15]. In contrast, SIRT1 aggravates cardiac hypertrophy by promoting membrane localization and activation of Akt and phosphatidylinositol-dependent protein kinase 1 [16]. These results imply a complex mechanism for SIRT1 in pathological cardiac hypertrophy.

Therefore, in this study, we investigated the regulatory mechanisms of UPRmt in pathological cardiac hypertrophy, to rescue damaged mitochondria from hypertrophy by regulating UPRmt. Based on previous studies, we identified the role of CTRP3 in regulating UPRmt and its mechanisms. The results of this study provide new ideas for treating pathological cardiac hypertrophy.

## Results

### CTRP3 overexpression alleviated pathological cardiac hypertrophy, mitochondrial dysfunction, and oxidative stress injury

We previously showed that CTRP3 protein levels were upregulated in transverse aortic constriction (TAC)-induced mouse hypertrophic hearts and exerted cardioprotective effects by inhibiting endoplasmic reticulum stress [9]. A pressure overload-induced cardiac hypertrophy model generated by TAC surgery and lentivirus overexpressing CTRP3 was used to study the role of CTRP3 in cardiac hypertrophy (Supplementary Fig. S1A). Echocardiographic analysis showed impaired cardiac function in TAC mice, as evidenced by reduced left ventricular ejection fraction (LVEF) and left ventricular fraction shortening (LVFS). However, CTRP3 overexpression in the myocardium partially restored TAC-induced cardiac systolic dysfunction (Supplementary Fig. S1B). Western blotting revealed increased β-MHC, ANP, collagen-3, and α-SMA protein levels, suggesting increased cardiac hypertrophy and fibrosis in TAC mice. Simultaneously, CTRP3 overexpression mitigated the increased protein levels of these molecules (Supplementary Fig. S1C). Overall, these results suggest that CTRP3 exerts anti-pathological effects on cardiac hypertrophy.

**Fig. 1:**
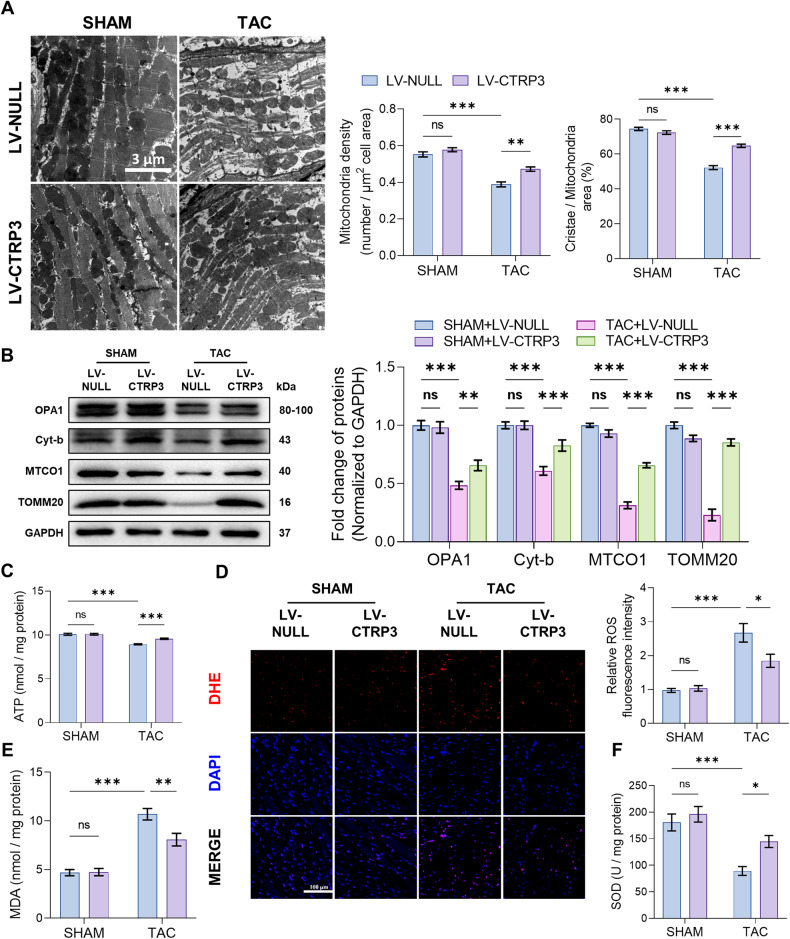
CTRP3 overexpression alleviated TAC-induced mitochondrial dysfunction and oxidative stress injury. **A** Left panel, representative electron microscope images of cardiomyocyte mitochondria. Right panel: Quantification of mitochondrial density and cristae/mitochondrial area in heart tissues (*n* = 5, 10–15 random fields were analyzed per sample). **B** Representative western blotting and quantification of OPA1, Cyt-b, MTCO1, and TOMM20 protein levels in heart tissues. GAPDH served as the loading control (*n* = 4). **C** Quantification of ATP levels in heart tissues (*n* = 5). **D** Left panel, representative fluorescence images of cardiac sections stained with DHE (red) to indicate ROS levels; nuclei were stained with DAPI (blue). Right panel, relative quantification of ROS fluorescence intensity in heart tissues (*n* = 6, 10–15 random fields were analyzed per sample). **E** Quantification of MDA levels in heart tissues (*n* = 10). **F** Quantification of SOD activity in heart tissues (*n* = 10). Data were analyzed by one-way ANOVA and represented as mean ± SEM. ^*^P < 0.05; ^**^P < 0.01; ^***^P < 0.001; ns not significant, MDA malondialdehyde, SOD superoxide dismutase, TAC transverse aortic constriction.

Next, we investigated the anti-hypertrophic effect of CTRP3 in relation to mitochondrial function and oxidative stress injury. The electron microscopy results reveal a significant decrease in mitochondria density and cristae in TAC mice, and that CTRP3 overexpression reduced TAC-induced mitochondrial damage (Fig. 1A). Western blotting revealed significantly reduced OPA1, Cyt-b, MTCO1, and TOMM20 protein levels in the hearts of TAC mice, and that CTRP3 overexpression partially upregulated the protein levels of these proteins. This indicates that CTRP3 overexpression had a protective effect against TAC-induced mitochondrial dysfunction (Fig. 1B). This was also demonstrated by the ATP content of the mouse heart under different treatments (Fig. 1C). The effect of CTRP3 on oxidative stress injury was examined given the substantial production of ROS during oxidative respiration in the mitochondria. Dihydroethidium (DHE) staining (Fig. 1D), malondialdehyde (MDA) levels (Fig. 1E), and superoxide dismutase (SOD) activity levels (Fig. 1F) showed that TAC-induced pressure overload increased ROS levels and lipid peroxidation toxicity, but decreased antioxidant enzyme activity. However, these responses were reversed by CTRP3 overexpression. The in vitro results also demonstrate that Ang II-induced neonatal rat cardiomyocytes (NRCMs) hypertrophy was accompanied by mitochondrial dysfunction and oxidative stress injury and that CTRP3 treatment ameliorated these pathological conditions (Supplementary Fig. S2). Collectively, these results suggest that CTRP3 exerts an anti-cardiac hypertrophic effect by improving mitochondrial function and reducing oxidative stress.

### CTRP3 regulated UPRmt under mitochondrial dysfunction and oxidative stress injury

**Fig. 2:**
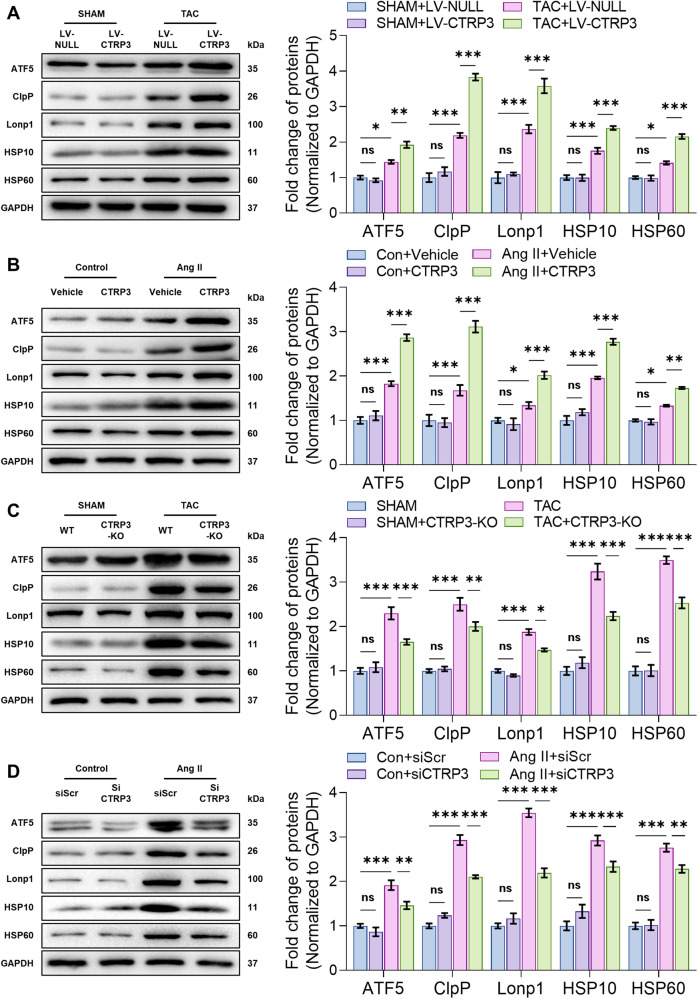
CTRP3 regulated UPRmt. **A**–**D** Representative western blotting and quantification of ATF5, ClpP, Lonp1, HSP10, and HSP60 protein levels in heart tissues (**A** and **C**) and NRCMs (**B** and **D**) in response to different treatments. GAPDH served as the loading control (*n* = 4). Data were analyzed by one-way ANOVA and presented as mean ± SEM. ^*^P < 0.05; ^**^P < 0.01; ^***^P < 0.001; ns not significant.

UPRmt is a crucial mechanism for maintaining mitochondrial protein homeostasis. We hypothesized that the process by which CTRP3 exerts its mitochondrial protective function also involves UPRmt. To investigate this, we investigated the UPRmt-related proteins (ATF5, ClpP, Lonp1, HSP10, and HSP60). CTRP3 overexpression under physiological conditions did not affect UPRmt. In contrast, TAC-induced mitochondrial dysfunction led to significantly increased UPRmt-related protein levels, whilst CTRP3 overexpression further activated TAC-induced UPRmt (Fig. 2A). Inducing NRCM hypertrophy using Ang II activated UPRmt in NRCMs, and CTRP3 overexpression further activated this response (Fig. 2B), which is consistent with the in vivo findings.

CTRP3 expression was inhibited in vivo and in vitro to test the effect of CTRP3 inhibition on UPRmt (Supplementary Fig. S3). Compared to wildtype (WT) mice, UPRmt-related protein levels were not altered significantly in the hearts of CTRP3-KO mice; however, TAC-induced UPRmt-related protein levels were inhibited in CTRP3-KO mice (Fig. 2C). Similarly, CTRP3 knockdown inhibited the Ang II-induced UPRmt-related protein levels in NRCMs (Fig. 2D). Collectively, these findings indicate that CTRP3 did not affect UPRmt under physiological conditions. However, during mitochondrial dysfunction and oxidative stress injury, CTRP3 overexpression activated UPRmt, whereas CTRP3 knockout or knockdown inhibited UPRmt activation. Mitochondrial dysfunction and oxidative stress injury activated UPRmt to maintain mitochondrial function, whilst CTRP3 was a key molecule in regulating this response.

### ATF5 knockout blocked the cardioprotective effect of CTRP3 in TAC mice

**Fig. 3:**
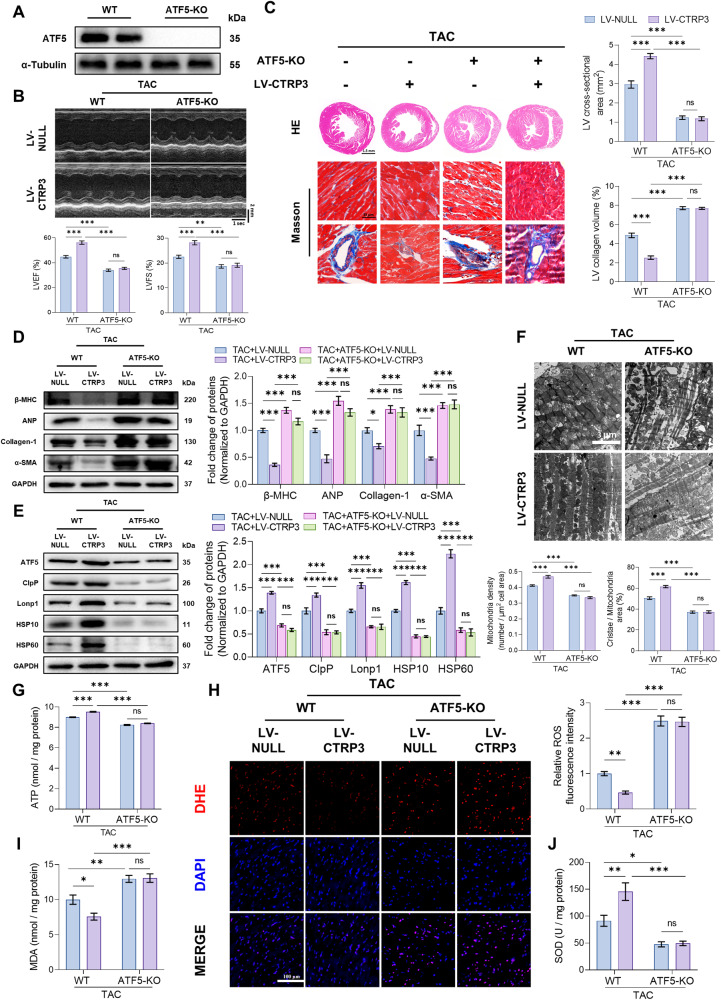
ATF5 knockout blocked the cardioprotective effect of CTRP3 in TAC mice. **A** Representative western blotting of ATF5 protein level in the heart tissues of ATF5-KO mice. α-Tubulin served as a loading control. **B** Top panel, representative echocardiographic images 4 weeks after TAC surgery. Bottom two panels, LVEF and LVFS determined by analyzing echocardiographic images (*n* = 10). **C** Left panel, representative images of heart sections stained with HE and Masson’s trichrome. Right panel, quantification of LV cross-sectional area and left ventricular collagen volume (*n* = 5, 10–15 random fields were analyzed per sample). **D** Representative western blotting and quantification of β-MHC, ANP, collagen-1, and α-SAM protein levels in heart tissues. GAPDH served as a loading control (*n* = 4). **E** Representative western blotting and quantification of ATF5, ClpP, Lonp1, HSP10, and HSP60 protein levels in heart tissues. GAPDH served as a loading control (*n* = 4). **F** Top panel, representative electron microscope images of cardiomyocyte mitochondria in heart tissues. Bottom two panels, quantification of mitochondria density, cristae/mitochondrial area in heart tissues (*n* = 5, 10–15 random fields were analyzed per sample). **G** Quantification of ATP levels in heart tissues (*n* = 5). **H** Left panel, representative fluorescence images of cardiac sections stained with DHE (red) to indicate ROS levels; nuclei were stained with DAPI (blue). Right panel, relative quantification of ROS fluorescence intensity in heart tissues (*n* = 6, 10–15 random fields were analyzed per sample). **I** Quantification of MDA levels in heart tissues (*n* = 10). **J** Quantification of SOD activities in heart tissues (*n* = 10). Data were analyzed by one-way ANOVA and presented as mean ± SEM. ^*^P < 0.05; ^**^P < 0.01; ^***^P < 0.001; ns not significant, HE hematoxylin and eosin, MDA malondialdehyde, SOD superoxide dismutase, TAC transverse aortic constriction.

ATF5 plays a key role in regulating UPRmt in mammalian cells [5]. To understand whether ATF5 mediated the role of CTRP3 in UPRmt, ATF5-KO mice were generated (Fig. 3A). Although neonatal ATF5-KO mice have a high mortality rate, studies have reported no significant difference in cardiac phenotype in adult ATF5-KO mice [17]. In our study, the cardiac function of adult ATF5-KO mice was comparable to that of WT mice, thus, we used the former in our study. As previously described, CTRP3 overexpression mitigated TAC-induced reduction in LVEF and LVFS and increased left ventricular collagen volume. However, ATF5 knockout prevented the protective effect of CTRP3 and led to a further decrease in LVEF and LVFS, as well as an increase in left ventricular collagen volume (Fig. 3B, C). In addition, β-MHC, ANP, collagen-3, and α-SMA protein levels were reduced in TAC mice after CTRP3 overexpression, whilst ATF5 knockout further increased these protein levels compared with those of TAC mice (Fig. 3D). These results suggest that CTRP3 overexpression improved cardiac hypertrophy and fibrosis, although ATF5 knockout blocked the protective effect of CTRP3 and exacerbated cardiac hypertrophy and fibrosis.

CTRP3 overexpression further induced UPRmt-related protein levels in TAC mice, however, ATF5 knockout significantly inhibited their levels (Fig. 3E). Electron microscopy subsequently showed that the damaged mitochondrial density and mitochondrial cristae in TAC mice improved with CTRP3 overexpression. However, improved mitochondrial morphology was not observed with ATF5 knockout, resulting in more severe damage (Fig. 3F). ATP content also varied alongside changes in mitochondrial morphology. These results show that ATF5 knockout reduced ATP content in the hearts of TAC mice whilst inhibiting increased ATP production following CTRP3 overexpression (Fig. 3G).

Finally, oxidative stress injuries were investigated. CTRP3 overexpression inhibited the increased oxidative stress injury induced by TAC, as evidenced by the observed decreases in ROS (Fig. 3H) and MDA levels (Fig. 3I) alongside increased SOD activity (Fig. 3J). However, ATF5 knockout hampered the effects of CTRP3 and increased oxidative stress. Collectively, these results suggest that CTRP3 induces UPRmt by increasing ATF5 protein levels, thereby improving mitochondrial dysfunction and oxidative stress injury.

### SIRT1 is a potential downstream molecule mediating the role of CTRP3

**Fig. 4:**
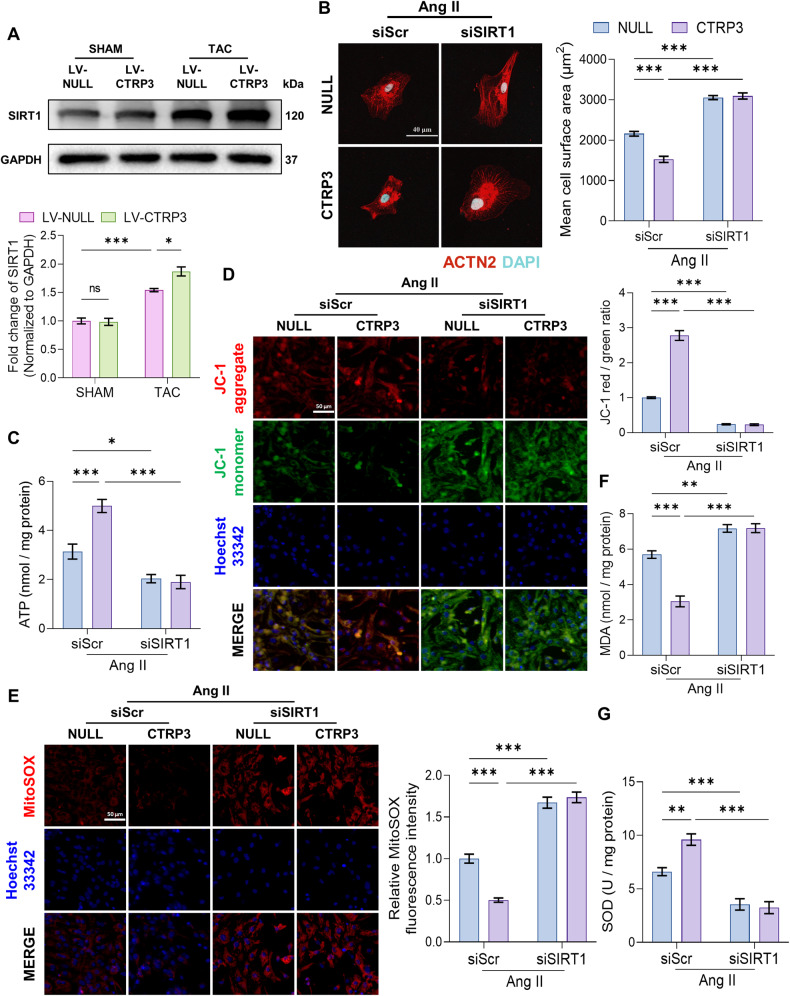
SIRT1 may be a downstream molecule to mediate the role of CTRP3. **A** Representative western blotting analysis and quantification of SIRT1 protein level in heart tissues. GAPDH served as the loading control (*n* = 4). **B** Left panel, representative fluorescence images of NRCMs stained with ACTN2 (red) and nuclei stained with DAPI (blue). Right panel, quantification of the mean cell surface area of NRCMs (*n* = 6; at least 30 random cells were analyzed per sample). **C** Quantification of ATP levels in NRCMs (*n* = 6). **D** Left panel, representative fluorescence images of mitochondrial transmembrane potential stained with JC-1 in NRCMs. Right panel, relative quantification of the ratio of JC-1 red/green fluorescence intensity in NRCMs (*n* = 6, 10–15 random fields were analyzed per sample). **E** Left panel, representative fluorescence images of NRCMs stained with MitoSOX Red (red) to indicate mitochondrial ROS levels. Right panel, relative quantification of mitochondrial ROS fluorescence intensity of NRCMs (*n* = 6, 10–15 random fields were analyzed per sample). **F** Quantification of MDA levels in NRCMs (*n* = 9). **G** Quantification of SOD activity in NRCMs (*n* = 9). Data were analyzed by one-way ANOVA and presented as mean ± SEM. ^*^P < 0.05; ^**^P < 0.01; ^***^P < 0.001; ns not significant, MDA malondialdehyde, NRCM neonatal rat cardiomyocyte.

SIRT1 can protect against pathological cardiac hypertrophy in addition to activating UPRmt [14, 15, 18, 19]. We found that SIRT1 protein levels increased in the hearts of TAC mice, which was further induced by CTRP3 treatment (Fig. 4A).

To elucidate the relationship between SIRT1 and CTRP3, SIRT1 protein levels were inhibited in NRCMs. CTRP3 treatment suppressed Ang II-induced NRCM hypertrophy. Conversely, SIRT1 knockdown resulted in severe NRCM hypertrophy, which was not rescued by CTRP3 treatment (Fig. 4B). In addition, CTRP3 treatment restored mitochondrial function in NRCMs, as evidenced by decreased ATP content alongside increased mitochondrial transmembrane potential and MitoTracker fluorescence intensity (Fig. 4C, D and Supplementary S4A). Moreover, CTRP3 treatment reduced mitochondrial ROS (Fig. 4E), MDA (Fig. 4F), and 8-oxoG levels (Supplementary Fig. S4B), which represent common types of DNA damage caused by ROS, in addition to increasing SOD activity (Fig. 4G), thereby reducing oxidative stress injury. However, SIRT1 knockdown completely reversed the protective effect of CTRP3 and aggravated Ang II-induced NRCM damage. Collectively, these results indicate that SIRT1 mediated the protective effects of CTRP3 in NRCMs.

### SIRT1 knockout blocked the cardioprotective effect of CTRP3 in TAC mice

**Fig. 5:**
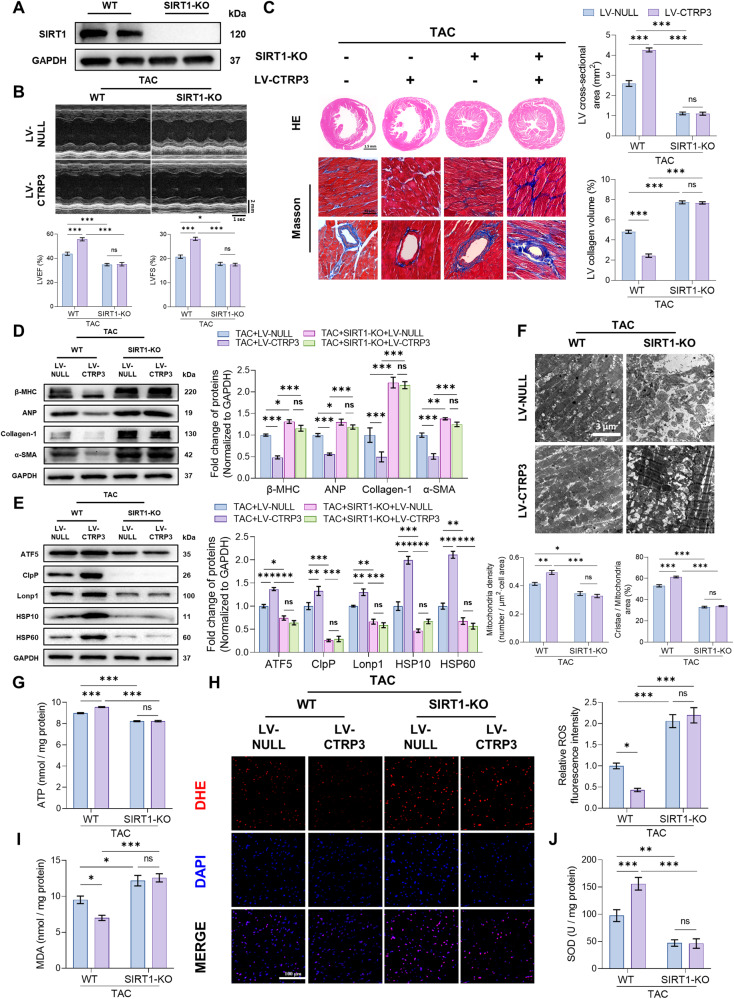
SIRT1 knockout blocked the cardioprotective effect of CTRP3 in TAC mice. **A** Representative western blotting of SIRT1 protein level in the heart tissues of SIRT1-KO mice. GAPDH serves as a loading control. **B** Top panel, representative echocardiographic images 4 weeks after TAC surgery. Bottom two panels, LVEF and LVFS determined by analyzing echocardiographic images (*n* = 10). **C** Left panel, representative images of heart sections stained with HE and Masson’s trichrome. Right panel, quantification of left ventricular cross-sectional area and collagen volume (*n* = 5, 10–15 random fields were analyzed per sample). **D** Representative western blotting and quantification of β-MHC, ANP, collagen-1, and α-SAM protein levels in heart tissues. GAPDH served as a loading control (*n* = 4). **E** Representative western blotting and quantification of ATF5, ClpP, Lonp1, HSP10, and HSP60 protein levels in heart tissues. GAPDH served as a loading control (*n* = 4). **F** Top panel, representative electron microscope images of a cardiomyocyte mitochondrion of the heart. Bottom two panels, quantification of mitochondria density, cristae/mitochondrial area in heart tissues (*n* = 5, 10–15 random fields were analyzed per sample). **G** Quantification of ATP levels in heart tissues (*n* = 5). **H** Left panel, representative fluorescence images of cardiac sections stained with DHE (red) to indicate ROS levels; nuclei were stained with DAPI (blue). Right panel, relative quantification of ROS fluorescence intensity in heart tissues (*n* = 6, 10–15 random fields were analyzed per sample). **I** Quantification of MDA levels in heart tissues (*n* = 10). **J** Quantification of SOD activities in heart tissues (*n* = 10). Data were analyzed by one-way ANOVA and presented as mean ± SEM. ^*^P < 0.05; ^**^P < 0.01; ^***^P < 0.001; ns not significant, LVEF left ventricular ejection fraction, LVFS left ventricular fraction shortening, MDA malondialdehyde, TAC transverse aortic constriction.

To better determine the role of SIRT1 in pathological cardiac hypertrophy, we knocked out SIRT1 expression in mice (Fig. 5A). CTRP3 overexpression reduced LVEF and LVFS, in addition to increasing the left ventricular collagen volume in TAC mice. In contrast, SIRT1 knockout prevented CTRP3 from rescuing these indices and even worsened them in some cases (Fig. 5B, C). Analysis of cardiac hypertrophy-related (β-MHC, ANP) and cardiac fibrosis-related (collagen-3, α-SMA) protein levels also supported this hypothesis (Fig. 5D).

Furthermore, SIRT1 knockout significantly inhibited UPRmt-related protein levels in TAC mice regardless of whether CTRP3 was overexpressed (Fig. 5E). Electron microscopy of mitochondria revealed increased mitochondrial density and cristae following CTRP3 overexpression. However, SIRT1 knockout strongly decreased mitochondrial density and mitochondrial cristae (Fig. 5F), with the same trend observed in the ATP content assays (Fig. 5G). Furthermore, SIRT1 knockout increased ROS (Fig. 5H) and MDA (Fig. 5I), while reducing SOD activity (Fig. 5J) to a greater extent than the effects of TAC-induced oxidative stress injury, and inhibited CTRP3 function. In summary, these results suggest that SIRT1 plays a key role in mediating the protective effects of CTRP3 against mitochondrial dysfunction and oxidative stress injury.

### CTRP3 functioned via the SIRT1/ATF5 axis to activate UPRmt

**Fig. 6:**
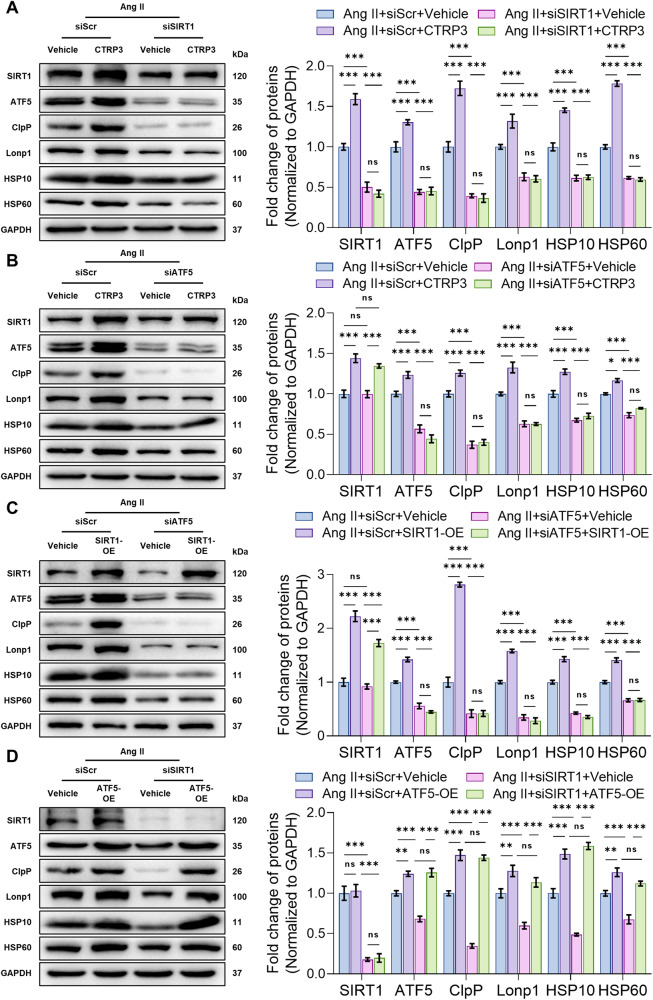
CTRP3 functioned via the SIRT1/ATF5 axis to activate UPRmt. **A**–**D** Representative western blotting analysis and quantification of SIRT1, ATF5, ClpP, Lonp1, HSP10, and HSP60 protein levels in NRCMs. GAPDH served as the loading control (*n* = 4). Data were analyzed by one-way ANOVA and presented as mean ± SEM. ^*^P < 0.05; ^**^P < 0.01; ^***^P < 0.001; ns not significant, NRCM neonatal rat cardiomyocyte.

**Fig. 7:**
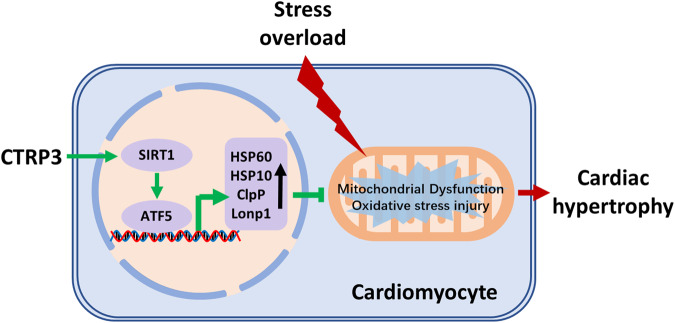
Schematic illustration. CTRP3 promotes the levels of UPRmt-related proteins through the SIRT1/ATF5 axis and attenuates stress overload-induced mitochondrial dysfunction and oxidative stress damage, thereby alleviating pathological cardiac hypertrophy.

Finally, the relationship between ATF5 and SIRT1 was investigated. ATF5 and SIRT1 protein levels were inhibited in vitro. Consistent with previous results, ATF5 or SIRT1 knockdown suppressed Ang II-induced UPRmt-related protein levels and inhibited UPRmt activation by CTRP3, demonstrating that both ATF5 and SIRT1 mediated the effects of CTRP3. Additionally, SIRT1 knockdown inhibited ATF5 protein levels, although ATF5 knockdown did not affect SIRT1 protein levels, thus suggesting that ATF5 is a downstream effector molecule of SIRT1 (Fig. 6A, B). Next, ATF5 knockdown concomitant with SIRT1 overexpression showed that SIRT1 overexpression promoted UPRmt-related protein levels. ATF5 knockdown also prevented UPRmt activation by SIRT1, although SIRT1 overexpression was not inhibited by ATF5 knockdown (Fig. 6C). Furthermore, simultaneous knockdown of SIRT1 and ATF5 expression in NRCMs showed that ATF5 overexpression rescued UPRmt inhibition by SIRT1 knockdown (Fig. 6D). Collectively, these results suggest that CTRP3 promoted UPRmt activation via the SIRT1/ATF5 axis, thereby combating mitochondrial dysfunction and oxidative stress injury (Fig. 7).

## Discussion

Pathological cardiac hypertrophy, an adaptive change in response to various stimuli, is caused by hypoxia, pressure or volume overload, metabolic disease, and genetics, among other factors [20]. Notably, pathological cardiac hypertrophy is an independent risk factor for heart failure, and the prolonged presence of these stimuli can worsen pathological cardiac hypertrophy [21]. Cardiomyocytes undergo apoptosis and necrosis due to chronic pathological stimuli, with cardiomyocyte condition and function deteriorating further and eventually leading to heart failure [22]. The long-term survival rate of patients with heart failure remains low. One recent study on the follow-up of hospitalized patients with heart failure reported that approximately 20% of patients die within two years of diagnosis due to heart failure exacerbation or sudden death [23]. Although heart transplantation and artificial hearts are effective treatments for heart failure, the availability of donors, difficulties with the procedures, and the overall costs have limited their application [24, 25]. Therefore, the molecular mechanisms involved in pathological cardiac hypertrophy must be further investigated to identify new targets for drug development as well as therapeutic strategies. Here, we identified a novel mechanism of CTRP3 against cardiac hypertrophy by activating UPRmt via the SIRT1/ATF5 axis.

Our research group has long focused on CTRP3 function in health and disease. Previous reports show that CTRP3 plays various roles in biological processes, such as inflammation, glycolipid metabolism, oxidative stress, and apoptosis [10, 26–28]. In the context of cardiovascular disease, CTRP3 can resist apoptosis and oxidative stress injury, and alleviate myocardial ischemia/reperfusion injury via the LAMP1/JIP2/JNK signaling pathway [29]. In addition, CTRP3 exerts protective effects against diabetic cardiomyopathy and adriamycin-induced cardiac injury [30, 31]. We previously demonstrated that CTRP3 reduces pathological cardiac hypertrophy by inhibiting the p38/CREB pathway and p38-induced endoplasmic reticulum stress [9]. We also reported reduced mitochondrial density and damaged mitochondrial cristae in TAC mice accompanied by decreased ATP production and increased oxidative stress injury. CTRP3 overexpression significantly improved mitochondrial function and oxidative stress injury in TAC mice. These findings provide new insights into the cardioprotective effects of CTRP3.

Pathological cardiac hypertrophy is accompanied by increased energy demand, but is inevitably followed by cell death, vascular damage, increased fibrosis, and metabolic dysfunction, ultimately resulting in damaged mitochondria that cannot meet these increased energy demands [20]. One key feature of mitochondrial damage is the disruption of mitochondrial protein homeostasis and the accumulation of unfolded or misfolded proteins within the mitochondria. Under these conditions, UPRmt is activated via mitochondria–nucleus communication. The levels of some proteins, such as proteases (for example, ClpP and Lonp1) and heat shock proteins (such as HSP60 and HSP10), can be induced to help correctly fold or degrade unfolded proteins and to restore mitochondrial protein homeostasis [4]. Our previous studies also identified substantially elevated mRNA levels of UPRmt-related genes in pressure overload-induced pathological hypertrophied hearts [32]. Furthermore, we observed UPRmt activation at the protein level in pathological cardiac hypertrophy. CTRP3 overexpression further increased URPmt-related protein levels in the hearts of TAC mice and attenuated TAC-induced pathological cardiac hypertrophy, mitochondrial dysfunction, and oxidative stress injury. Based on our previous findings, we conclude that the disruption of mitochondrial protein homeostasis and increased unfolded proteins in pathological cardiac hypertrophy induce a protective regulatory mechanism, UPRmt, to reduce mitochondrial damage and maintain cardiac function. Although this compensatory effect is limited, CTRP3 overexpression further activated UPRmt and strongly contributed to mitochondrial repair and recovery of cardiac function. Studies have reported that excessive UPRmt activation can be detrimental; therefore, precise detection and regulation of UPRmt should be the focus of future research [33, 34].

Earlier mechanistic studies revealed that UPRmt in nematodes is primarily regulated by the transcription factor, ATFS-1 [35]. Subsequent studies showed that ATF5, the homeotic gene of ATFS-1, regulated UPRmt in mammalian cells and induced high UPRmt-related protein levels, including HSP60 and Lonp1 [5]. This study found that ATF5 knockout inhibited CTP3-mediated UPRmt activation, and the cardioprotective effect of CTRP3 was severely impaired. These results provide evidence that ATF5 is a potential key molecule that exerts a cardioprotective effect on CTRP3 via UPRmt. The activation of UPRmt is also influenced by other molecules, such as CHOP and ERα [36, 37]. The association of UPRmt activation by CTRP3 with other pathways is not clear. We believe that the mechanisms involved are quite complex, and exploring this aspect may be a direction for us to continue our research in the future.

SIRT1, a NAD-dependent protein deacetylase, has been extensively studied in the cardiovascular field. SIRT1 resists oxidative stress injury through AMPK, Nrf2, NF-κB, p53, and other molecules or signaling pathways whilst also playing a protective role in various cardiovascular diseases, such as hypertension, cardiac hypertrophy, atherosclerosis, and ischemia/reperfusion injury [13]. SIRT1 is involved in mitochondrial biogenesis via PGC-1α and it regulates mitochondrial autophagy [38–42]. SIRT1 can activate UPRmt in *Caenorhabditis elegans*, zebrafish, and mouse lungs and adipocytes [19, 43–45]. In our study, SIRT1 knockout inhibited UPRmt activation, thus further worsening TAC-induced mitochondrial dysfunction and oxidative stress injury. In vivo, we observed that SIRT1 may be an upstream molecule that activates ATF5.

The most notable limitation of our study was the use of global knockout mice rather than heart-specific knockout mice. However, we did not find substantial cardiac abnormalities in these knockout mice. Various studies have identified detrimental phenotypes in knockout mice (Supplementary Table 1); for example, ATF5-KO mice may have abnormal cortical development and abnormal behaviors, such as anxiety, and SIRT1-KO mice may have abnormal bone development, autoimmune disease, and other problems [46–50]. As SIRT1 and ATF5 are widely distributed in vivo and play key roles in different tissues, our results may have been affected by other tissues or organs. This should be investigated in depth, which may require the use of cardiac-specific knockout mice. The other limitation of this study is that the direct or indirect interaction between SIRT1 and ATF5 was not confirmed. We previously showed that partial UPRmt activation by the PGC-1α/ATF5 axis mediated the cardioprotective effects of tetrahydrocurcumin [32]. We, therefore, hypothesize that communication between SIRT1 and ATF5 may involve PGC-1α, although this remains to be verified experimentally. Furthermore, we have not studied the function of mitochondria thoroughly. In future studies, we are considering experiments such as extracting mitochondria alone and measuring cellular oxygen consumption, which could make our conclusions more robust.

Finally, another recent study reported substantially lower levels of CTRP3 mRNA in the epicardial fat of patients with coronary artery disease combined with type 2 diabetes, which may be associated with increased inflammatory factors alongside an increased risk of coronary atherosclerosis [51]. Notably, mice do not have much epicardial fat, and anatomical differences may lead to a completely different pathophysiological process [52]. In addition, CTRP3 is reportedly correlated with obesity, diabetes, and inflammation, among other factors, although most clinical trials have only conducted correlational studies [53–57]. Furthermore, more studies are warranted before the anti-cardiac hypertrophy effects of CTRP3 can be translated into clinical use.

In conclusion, this study shows that CTRP3 activated UPRmt through the SIRT1/ATF5 axis and alleviated mitochondrial dysfunction and oxidative stress injury under pathological cardiac hypertrophy. These findings have important implications for understanding the functional mechanisms of CTRP3, and contribute to the mechanistic understanding of UPRmt. Overall, these results suggest that targeting CTRP3 and UPRmt may be a novel strategy for the prevention and treatment of pathological cardiac hypertrophy.

## Materials and methods

### Experimental animals and treatment

All animal experiments were approved by the Air Force Military Medical University Committee on Animal Care and followed the Care and Use of Laboratory Animals of the Chinese Animal Welfare Committee. For this study, 8-week-old to 10-week-old male mice and one to three-day-old neonatal rats were obtained from the Experimental Animal Center of Air Force Military Medical University. Transgenic mice with global Ctrp3 (CTRP3-KO), Atf5 (ATF5-KO), and Sirt1 knockouts (SIRT1-KO) were obtained from the Nanjing Biomedical Research Institute of Nanjing University (Nanjing, China) and maintained as previously described [7]. The construction of CTRP3-KO and ATF5-KO mice was performed on a C57BL/6J background using a clustered regularly interspaced short palindromic repeats (CRISPR)/Cas9 system. SIRT1-KO mice had a mixed 129/sv-CD1 genetic background. Due to the higher mortality of ATF5-KO mammary mice, each group did not necessarily comprise the same sex or litter, but mice were matched for sex and age between groups. Sample size in animal experiments ensures the reliability and validity of the results while conforming to the 3R principles. Animals were randomly assigned to different groups using the random number table method. Researchers were not blinded to the animal grouping.

To overexpress CTRP3 in the hearts of mice, intramyocardial lentiviral injections were performed as described previously [7]. A lentivirus overexpressing Ctrp3 (LV-CTRP3; NM_001204134) was constructed by Shanghai Genechem Co., Ltd. (Shanghai, China). Next, 6-week-old to 8-week-old WT/transgenic mice were anesthetized using 1–2% isoflurane, and the toes were stimulated with no response, which confirmed successful anesthesia. Lentivirus was injected intramyocardially into the anterior, posterior, and lateral regions of the left ventricle (8–9 μL/spot, 1.2 ×10^12^ vg/mL). TAC or sham surgery was performed 2 weeks after the injection.

TAC surgery was subsequently performed to establish a murine model of chronic cardiac hypertrophy as described previously [7]. After administering anesthesia using 1–2% isoflurane, the mice were intubated with a ventilator (Minivent 845, Harvard Apparatus, USA). Next, the aortic arch was ligated using a 27 g needle and a pre-placed 6-0 thread between the cephalic trunk and the left common carotid artery, after which the needle was carefully withdrawn. The sham-operated mice underwent the same surgical procedure, except for aorta ligation.

### Echocardiography

M-mode echocardiography was performed using a Vevo 2100 small animal echo system (Visual Sonic, Canada) 4 weeks after TAC or sham surgery, as described previously [7]. Skin from the neck and chest of the mice was depilated. The mice were anesthetized using 1–2% isoflurane and the toes were stimulated without response. The anesthetized mice were secured to the working table, and the isoflurane concentration was adjusted to maintain the heart rate at 400–500 bpm. The parasternal short-axis view of the left ventricle was recorded and then measured using Vevo Lab 5.6.0 software (Visual Sonic, Canada). The LVEF and LVFS were calculated automatically using the same software.

### Hematoxylin-eosin (HE) and Masson’s trichrome staining

Four weeks after TAC or sham surgery, the mouse hearts were harvested, as described previously, before fixation in 4% paraformaldehyde for at least 24 h [7]. The fixed hearts were cut into 5-μm-thick sections from the cardiac apex and subjected to HE or Masson’s trichrome staining (Servicebio, Wuhan, China). The sections were scanned using a Pannoramic MIDI scanner (3DHISTECH, Hungary), while the collagen volume (%), which represented the percentage of collagen in the total area, was quantified using ImageJ software (NIH, USA).

### Transmission electron microscopy

Heart tissues were fixed using a commercial electron microscope fixative (G1102, Servicebio). After dehydration, thin sections were stained with lead citrate and uranyl acetate. Images were captured using a JEM-1400 transmission electron microscope (JEOL, Tokyo, Japan). To quantify the mitochondrial quality, mitochondrial density, which represents the ratio of the mitochondrial number to the total image area, and cristae/mitochondrial area (%), which represents the average percentage of cristae in the mitochondrial area, were calculated using ImageJ software (NIH).

### Western blotting

Western blotting was performed as described previously [7]. Briefly, total protein was extracted from the left ventricles of mice and NRCMs with RIPA lysis buffer (P0013C, Beyotime, China). An Enhanced BCA Protein Assay Kit (P0009, Beyotime) was used to quantify the protein content, according to the manufacturer’s instructions. Subsequently, 10–30 μg protein from each sample was separated by sodium dodecyl sulfate-polyacrylamide gel electrophoresis and then transferred to polyvinylidene fluoride membranes (ISEQ00010, Millipore, USA). The membranes were blocked with 5% skim milk dissolved in TBST for 1 h at room temperature. Membranes were then incubated with the following diluted primary antibodies: anti-SIRT1 (1:1000, 21535-1-AP), anti-β-MHC (1:1000, 22280-1-AP), anti-ANP (1:1000, 27426-1-AP), anti-collagen-3 (1:1000, 22734-1-AP), anti-Cyt-b (1:1000, 55090-1-AP), anti-TOMM20 (1:1000, 11802-1-AP), anti-ClpP (1:1000, 15698-1-AP), and anti-Lonp1 (1:1000, 15440-1-AP) purchased from Proteintech (Wuhan, China); anti-α-SMA (1:5000, ab7817), anti-MTCO1 (1:1000, ab14705), and anti-ATF5 (1:2000, ab184923) purchased from ABCAM (Cambridge, UK); anti-GAPDH (1:5000, AT0002) purchased from Engibody (WI, USA); anti-HSP10 (1:1000, A5580) purchased from ABclonal (Wuhan, China); anti-HSP60 (1:1000, 4870s) purchased from CST (MA, USA); and anti-OPA1(1:1000, 612606) purchased from BD Biosciences (NY, USA) at 4 °C overnight. The membranes were then washed with TBST and incubated with HRP-conjugated goat anti-rabbit IgG (1:5000, ZB-2301) or HRP-conjugated goat anti-mouse IgG (1:5000, ZB-2305; Zsbio, Beijing, China) for 2 h at room temperature. The membranes were then incubated with an ECL reagent (WBULS0500, Millipore, USA) and scanned using a ChemiDoc™ XRS+ System (Bio-Rad, USA). The gray values of the protein bands were analyzed using ImageJ software (NIH).

### ROS detection

To visualize the ROS levels within heart tissues 4 weeks after TAC or sham surgery, frozen sections of heart tissue were prepared, as described previously, then stained with the superoxide anion fluorescence probe DHE (S0063, Beyotime) for 30 min at 37 °C [29].

The global or mitochondrial ROS levels of NRCMs were detected using a ROS Assay Kit (S0033S, Beyotime) or MitoSOX Red (M36008, Invitrogen, USA). After treatment, NRCMs were incubated with serum-free DMEM/F-12 culture medium containing 10 μM dichloro-dihydro-fluorescein diacetate (DCFH-DA) or 5 μM MitoSOX Red for 30 min at 37 °C. After staining was complete, the medium was replaced with fresh pre-warmed medium, and NRCMs were observed immediately using an LSM900 confocal microscope (Carl Zeiss, Germany). ImageJ software (NIH) was used to measure the fluorescence intensity.

### ATP, MDA, and SOD detection

Heart tissue and NRCMs were lysed according to the manufacturer’s instructions, and the protein content was quantified using an Enhanced BCA Protein Assay Kit (P0009, Beyotime). Furthermore, ATP, MDA content, and SOD activities were measured using the Enhanced ATP Assay Kit (S0027, Beyotime), Lipid Peroxidation MDA Assay Kit (S0131, Beyotime), and Total Superoxide Dismutase Assay Kit (S0101, Beyotime), respectively, and were normalized to the total protein content of the sample.

### NRCM isolation and treatment

NRCMs were extracted and cultured as described previously [7]. In brief, the ventricles of neonatal rats were removed and placed in cold phosphate-buffered saline to wash out residual blood. All heart tissues were cut into pieces and mixed with 5 ml 0.4% trypsin (1004, BioFroxx, Germany) then incubated at 37 °C for 5 min. Next, the supernatant was removed, and the remaining tissue was digested using 0.1% collagenase II (2275, BioFroxx). Then, the cell suspension was centrifuged at 1000 rpm for 3 min and the cell pellet was resuspended in complete DMEM/F12 medium (PM150312, Pricella, China) containing 10% FBS (04-001-1ACS, BioInd, Israel), 0.1 mM BrdU (B8010, Solarbio, China), and 1% penicillin/streptomycin (P1400, Solarbio). The cell suspension was filtered through a 70-μm filter before cells were placed into culture flasks and incubated for 2 h at 37 °C. The culture supernatant, which mainly contained cardiomyocytes, was then collected. After counting, the cells were placed in 6-well plates (1 ×10^4^–10^5^/cm^2^) and after 2 d the medium was replaced with fresh complete DMEM/F12 medium.

Small interfering RNA (siRNA, A10001) targeting CTRP3, ATF5, and SIRT1, as well as corresponding overexpression plasmids (C05003) were constructed by GenePharma (Shanghai, China). Briefly, 100 pmol siRNA or 2 μg overexpression plasmid was transfected with Lipofectamine 3000 (L3000008, Invitrogen) according to the manufacturer’s instructions to knockdown or overexpress the target gene, respectively; the culture medium was changed after 4–6 h. After 24 h of transfection, NRCMs were stimulated with Ang II (1 μM) for 24 h to induce hypertrophy and/or were treated with CTRP3 (10 μg/mL) to observe the effect of CTRP3. These NRCMs were subsequently used in other experiments, as described below.

### Immunofluorescence staining

Immunofluorescence staining was performed as previously described [7]. NRCMs were fixed with 4% paraformaldehyde for 20 min and permeabilized with 0.3% Triton-X for 10 min at room temperature. After washing with phosphate-buffered saline, NRCMs were blocked with QuickBlock™ Blocking Buffer (P0260, Beyotime) for 15 min at room temperature, then incubated with anti-ACTN2 antibody (1:200, A7732, Sigma-Aldrich, USA) or anti-8-oxo guanine (8-oxoG, 1:50, sc-130914, Santa Cruz, USA) at 4 °C overnight. The next day, NRCMs were stained with Alexa Fluor 568-conjugated goat anti-mouse IgG (1:500, ab175473, ABCAM) for 2 h followed by a 5 min incubation with 4’,6-diamidino-2-phenylindole (DAPI; DA0002, Leagene, China) at room temperature. Finally, images were captured with an LSM900 confocal microscope (Carl Zeiss), and the NRCM size was calculated using ImageJ software (NIH).

### Detection of mitochondrial transmembrane potential

The mitochondrial transmembrane potential was detected using the Enhanced Mitochondrial Membrane Potential Assay Kit with JC-1 (P0009, Beyotime). After treatment, NRCMs were incubated with JC-1 working solution for 20 min at 37 °C according to the manufacturer’s instructions. After incubation, the cells were washed with buffer and subsequently observed under an LSM900 confocal microscope (Carl Zeiss). ImageJ software (NIH) was used to measure JC-1 red or green fluorescence intensity. The mitochondrial transmembrane potential level is represented by the JC-1 red/green ratio, which was calculated as the ratio of average fluorescence intensity of the JC-1 red/green.

### Statistical analysis

Statistical analysis was performed using GraphPad Prism (version 9.0; GraphPad Software, USA). One-way ANOVA was used to compare the differences between multiple groups. The homogeneity of variance was tested by the Levenes test. All experimental data are presented as mean ± SEM, and differences were considered statistically significant at *P* < 0.05.

### Supplementary information

Supplementary information

Original Data File

## Data Availability

All datasets generated and analyzed during this study are included in this published article and its Supplementary Information files. Additional data are available from the corresponding author on reasonable request.

## References

[1] Schiattarella GG, Hill JA (2015). Inhibition of hypertrophy is a good therapeutic strategy in ventricular pressure overload. Circulation.

[2] Rashid AM, Khan MS, Fudim M, DeWald TA, DeVore A, Butler J (2023). Management of heart failure with reduced ejection fraction. Curr Probl Cardiol.

[3] Quiros PM, Mottis A, Auwerx J (2016). Mitonuclear communication in homeostasis and stress. Nat Rev Mol Cell Biol.

[4] Roca-Portoles A, Tait SWG (2021). Mitochondrial quality control: from molecule to organelle. Cell Mol Life Sci.

[5] Fiorese CJ, Schulz AM, Lin YF, Rosin N, Pellegrino MW, Haynes CM (2016). The transcription factor ATF5 mediates a mammalian mitochondrial UPR. Curr Biol.

[6] Tan K, Fujimoto M, Takii R, Takaki E, Hayashida N, Nakai A (2015). Mitochondrial SSBP1 protects cells from proteotoxic stresses by potentiating stress-induced HSF1 transcriptional activity. Nat Commun.

[7] Kong M, Gao Y, Guo X, Xie Y, Yu Y (2021). Role of the CTRP family in tumor development and progression. Oncol Lett.

[8] Guo B, Zhuang T, Xu F, Lin X, Li F, Shan SK (2020). New Insights Into Implications of CTRP3 in Obesity, Metabolic Dysfunction, and Cardiovascular Diseases: Potential of Therapeutic Interventions. Front Physiol.

[9] Zhang B, Zhang P, Tan Y, Feng P, Zhang Z, Liang H (2019). C1q-TNF-related protein-3 attenuates pressure overload-induced cardiac hypertrophy by suppressing the p38/CREB pathway and p38-induced ER stress. Cell Death Dis.

[10] Zeng X, Peng Y, Wang Y, Kang K (2022). C1q/tumor necrosis factor-related protein-3 (CTRP3) activated by forkhead box O4 (FOXO4) down-regulation protects retinal pericytes against high glucose-induced oxidative damage through nuclear factor erythroid 2-related factor 2 (Nrf2)/Nuclear factor-kappaB (NF-κB) signaling. Bioengineered.

[11] Wang F, Zhao L, Shan Y, Li R, Qin G (2019). CTRP3 protects against high glucose-induced cell injury in human umbilical vein endothelial cells. Anal Cell Pathol.

[12] Lopaschuk GD, Karwi QG, Tian R, Wende AR, Abel ED (2021). Cardiac energy metabolism in heart failure. Circ Res.

[13] Wu QJ, Zhang TN, Chen HH, Yu XF, Lv JL, Liu YY (2022). The sirtuin family in health and disease. Signal Transduct Target Ther.

[14] Wang W, Wang L, Yang M, Wu C, Lan R, Wang W (2021). Circ-SIRT1 inhibits cardiac hypertrophy via activating SIRT1 to promote autophagy. Cell Death Dis.

[15] Shen T, Ding L, Ruan Y, Qin W, Lin Y, Xi C (2014). SIRT1 functions as an important regulator of estrogen-mediated cardiomyocyte protection in angiotensin II-induced heart hypertrophy. Oxid Med Cell Longev.

[16] Sundaresan NR, Pillai VB, Wolfgeher D, Samant S, Vasudevan P, Parekh V (2011). The deacetylase SIRT1 promotes membrane localization and activation of Akt and PDK1 during tumorigenesis and cardiac hypertrophy. Sci Signal.

[17] Wang YT, Lim Y, McCall MN, Huang KT, Haynes CM, Nehrke K (2019). Cardioprotection by the mitochondrial unfolded protein response requires ATF5. Am J Physiol Heart Circ Physiol.

[18] Li J, Huang J, Lu J, Guo Z, Li Z, Gao H (2019). Sirtuin 1 represses PKC-ζ activity through regulating interplay of acetylation and phosphorylation in cardiac hypertrophy. Br J Pharmacol.

[19] Du SH, Shi J, Yu TY, Hu XX, He SM, Cao YY (2022). Nicotinamide mononucleotide ameliorates acute lung injury by inducing mitonuclear protein imbalance and activating the UPRmt. Exp Biol Med.

[20] Nakamura M, Sadoshima J (2018). Mechanisms of physiological and pathological cardiac hypertrophy. Nat Rev Cardiol.

[21] Bernardo BC, Weeks KL, Pretorius L, McMullen JR (2010). Molecular distinction between physiological and pathological cardiac hypertrophy: experimental findings and therapeutic strategies. Pharmacol Ther.

[22] Bishop SP, Zhang J, Ye L (2022). Cardiomyocyte proliferation from fetal- to adult- and from normal- to hypertrophy and failing hearts. Biology.

[23] Zhang Y, Zhang R, An T, Huang Y, Guo X, Yin S (2015). The utility of galectin-3 for predicting cause-specific death in hospitalized patients with heart failure. J Card Fail.

[24] Varshney AS, DeFilippis EM, Cowger JA, Netuka I, Pinney SP, Givertz MM (2022). Trends and outcomes of left ventricular assist device therapy: JACC Focus Seminar. J Am Coll Cardiol.

[25] Guglin M, Zucker MJ, Borlaug BA, Breen E, Cleveland J, Johnson MR (2020). Evaluation for heart transplantation and LVAD implantation: JACC Council Perspectives. J Am Coll Cardiol.

[26] Zhang Y, Xu G, Huang B, Chen D, Ye R (2022). Astragaloside IV regulates insulin resistance and inflammatory response of adipocytes via modulating CTRP3 and PI3K/AKT signaling. Diabetes Ther.

[27] Yang Y, Li Y, Ma Z, Jiang S, Fan C, Hu W (2016). A brief glimpse at CTRP3 and CTRP9 in lipid metabolism and cardiovascular protection. Prog Lipid Res.

[28] Mei M, Qu LH, Cong X, Zhang Y, Xiang RL, Yu GY (2021). CTRP3 promotes TNF-α-induced apoptosis and barrier dysfunction in salivary epithelial cells. Cell Signal.

[29] Song Y, Zhang Y, Wan Z, Pan J, Gao F, Li F (2022). CTRP3 alleviates cardiac ischemia/reperfusion injury via LAMP1/JIP2/JNK signaling pathway. Aging.

[30] Ma ZG, Yuan YP, Xu SC, Wei WY, Xu CR, Zhang X (2017). CTRP3 attenuates cardiac dysfunction, inflammation, oxidative stress and cell death in diabetic cardiomyopathy in rats. Diabetologia.

[31] Yuan YP, Ma ZG, Zhang X, Xu SC, Zeng XF, Yang Z (2018). CTRP3 protected against doxorubicin-induced cardiac dysfunction, inflammation and cell death via activation of Sirt1. J Mol Cell Cardiol.

[32] Zhang B, Tan Y, Zhang Z, Feng P, Ding W, Wang Q (2020). Novel PGC-1α/ATF5 axis partly activates UPRmt and mediates cardioprotective role of tetrahydrocurcumin in pathological cardiac hypertrophy. Oxid Med Cell Longev.

[33] Zhao Y, Li HX, Luo Y, Cui JG, Talukder M, Li JL (2022). Lycopene mitigates DEHP-induced hepatic mitochondrial quality control disorder via regulating SIRT1/PINK1/mitophagy axis and mitochondrial unfolded protein response. Environ Pollut.

[34] Pena S, Sherman T, Brookes PS, Nehrke K (2016). The mitochondrial unfolded protein response protects against anoxia in Caenorhabditis elegans. PLoS One.

[35] Nargund AM, Fiorese CJ, Pellegrino MW, Deng P, Haynes CM (2015). Mitochondrial and nuclear accumulation of the transcription factor ATFS-1 promotes OXPHOS recovery during the UPR(mt). Mol Cell.

[36] Kumar M, Sharma S, Haque M, Kumar J, Hathi UPS, Mazumder S (2022). TLR22-induced pro-apoptotic mtROS Abets UPR(mt)-mediated mitochondrial fission in Aeromonas hydrophila-infected headkidney macrophages of Clarias gariepinus. Front Immunol.

[37] Riar AK, Burstein SR, Palomo GM, Arreguin A, Manfredi G, Germain D (2017). Sex specific activation of the ERalpha axis of the mitochondrial UPR (UPRmt) in the G93A-SOD1 mouse model of familial ALS. Hum Mol Genet.

[38] Wang J, Li S, Wang J, Wu F, Chen Y, Zhang H (2020). Spermidine alleviates cardiac aging by improving mitochondrial biogenesis and function. Aging.

[39] Jiang Y, Chen D, Gong Q, Xu Q, Pan D, Lu F (2021). Elucidation of SIRT-1/PGC-1α-associated mitochondrial dysfunction and autophagy in nonalcoholic fatty liver disease. Lipids Health Dis.

[40] Zhang C-L, Feng H, Li L, Wang J-Y, Wu D, Hao Y-T (2017). Globular CTRP3 promotes mitochondrial biogenesis in cardiomyocytes through AMPK/PGC-1α pathway. Biochim Biophys Acta Gen Subj.

[41] Gao J, Qian T, Wang W (2020). CTRP3 activates the AMPK/SIRT1-PGC-1α pathway to protect mitochondrial biogenesis and functions in cerebral ischemic stroke. Neurochem Res.

[42] Feng H, Wang JY, Zheng M, Zhang CL, An YM, Li L (2016). CTRP3 promotes energy production by inducing mitochondrial ROS and up-expression of PGC-1α in vascular smooth muscle cells. Exp Cell Res.

[43] Mouchiroud L, Houtkooper RH, Moullan N, Katsyuba E, Ryu D, Canto C (2013). The NAD(+)/Sirtuin pathway modulates longevity through activation of mitochondrial UPR and FOXO signaling. Cell.

[44] Lin YF, Sam J, Evans T (2021). Sirt1 promotes tissue regeneration in zebrafish through regulating the mitochondrial unfolded protein response. iScience.

[45] Zhang P, Konja D, Zhang Y, Xu A, Lee IK, Jeon JH (2022). Clusterin is involved in mediating the metabolic function of adipose SIRT1. iScience.

[46] Umemura M, Kaneko Y, Tanabe R, Takahashi Y (2021). ATF5 deficiency causes abnormal cortical development. Sci Rep.

[47] Umemura M, Ogura T, Matsuzaki A, Nakano H, Takao K, Miyakawa T (2017). Comprehensive behavioral analysis of activating transcription factor 5-deficient mice. Front Behav Neurosci.

[48] Zainabadi K, Liu CJ, Caldwell ALM, Guarente L (2017). SIRT1 is a positive regulator of in vivo bone mass and a therapeutic target for osteoporosis. PLoS One.

[49] Li X, Zhang S, Blander G, Tse JG, Krieger M, Guarente L (2007). SIRT1 deacetylates and positively regulates the nuclear receptor LXR. Mol Cell.

[50] Sequeira J, Boily G, Bazinet S, Saliba S, He X, Jardine K (2008). sirt1-null mice develop an autoimmune-like condition. Exp Cell Res.

[51] Matloch Z, Mraz M, Kasperova BJ, Kratochvilova H, Svoboda P, Pleyerova I (2022). Decreased epicardial CTRP3 mRNA levels in patients with type 2 diabetes mellitus and coronary artery disease undergoing elective cardiac surgery: a possible association with coronary atherosclerosis. Int J Mol Sci.

[52] Marchington JM, Mattacks CA, Pond CM (1989). Adipose tissue in the mammalian heart and pericardium: structure, foetal development and biochemical properties. Comp Biochem Physiol B.

[53] Barbieri D, Goicoechea M, Verde E, Garcia-Prieto A, Verdalles U, Perez de Jose A (2023). Obesity, chronic kidney disease progression and the role of the adipokine C1q/TNF related protein-3. Nefrologia.

[54] Choi KM, Hwang SY, Hong HC, Yang SJ, Choi HY, Yoo HJ (2012). C1q/TNF-related protein-3 (CTRP-3) and pigment epithelium-derived factor (PEDF) concentrations in patients with type 2 diabetes and metabolic syndrome. Diabetes.

[55] Fan W, Si Y, Xing E, Feng Z, Ding Z, Liu Y (2023). Human epicardial adipose tissue inflammation correlates with coronary artery disease. Cytokine.

[56] Kon M, Tanimura Y (2023). Responses of complement C1q/tumor necrosis factor-related proteins to acute aerobic exercise. Cytokine.

[57] Cordeiro AV, Bricola RS, Braga RR, Lenhare L, Silva VRR, Anaruma CP (2020). Aerobic exercise training induces the mitonuclear imbalance and UPRmt in the skeletal muscle of aged mice. J Gerontol A Biol Sci Med Sci.

