# Supplementary material for: CTRP3 alleviates mitochondrial dysfunction and oxidative stress injury in pathological cardiac hypertrophy by activating UPRmt via the SIRT1/ATF5 axis
Source: Cell Death Discov. 2024 Jan 26;10:53. doi: 10.1038/s41420-024-01813-x (PMC10817931; doi:10.1038/s41420-024-01813-x)
Supplement: Supplementary file 1 — Supplementary information [file 41420_2024_1813_MOESM1_ESM.pdf]

SUPPLEMENTARY FIGURES AND TABLE

Fig. S1

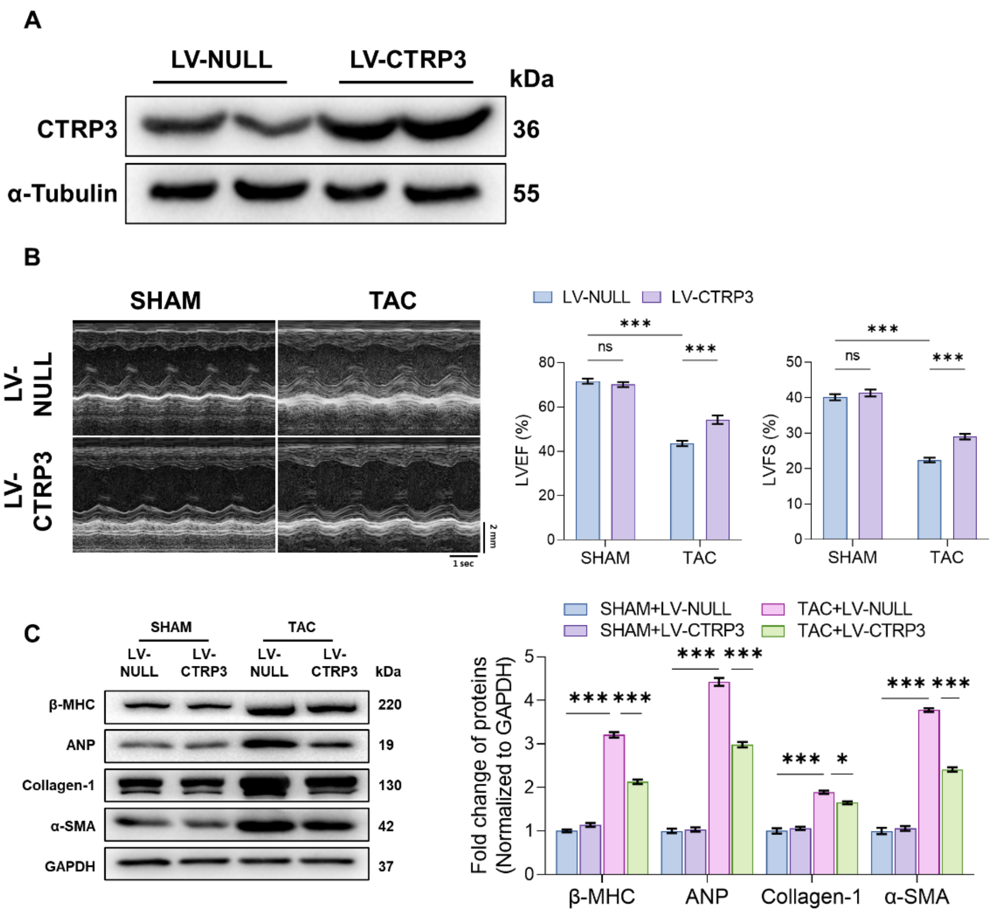

Fig. S1 CTRP3 overexpression alleviates TAC-induced pathological cardiac hypertrophy. A Representative western blotting analysis of CTRP3 levels in heart tissues after intramyocardial lentivirus injections. α-Tubulin was used as the loading control. **B** Left panel: representative echocardiographic images 4 weeks after TAC or sham surgery. Right two panels, LVEF and LVFS determined by analyzing the echocardiographic images (n = 10). **C** Representative western blotting analysis and quantification of β-MHC, ANP, Collagen-1, and α-SAM levels in the heart tissues. GAPDH served as the loading control (n = 4). Data were analyzed by one-way ANOVA and presented as mean ± SEM. $^{*}P < 0.05$; $^{***}P < 0.001$; ns, not significant.

Fig. S2

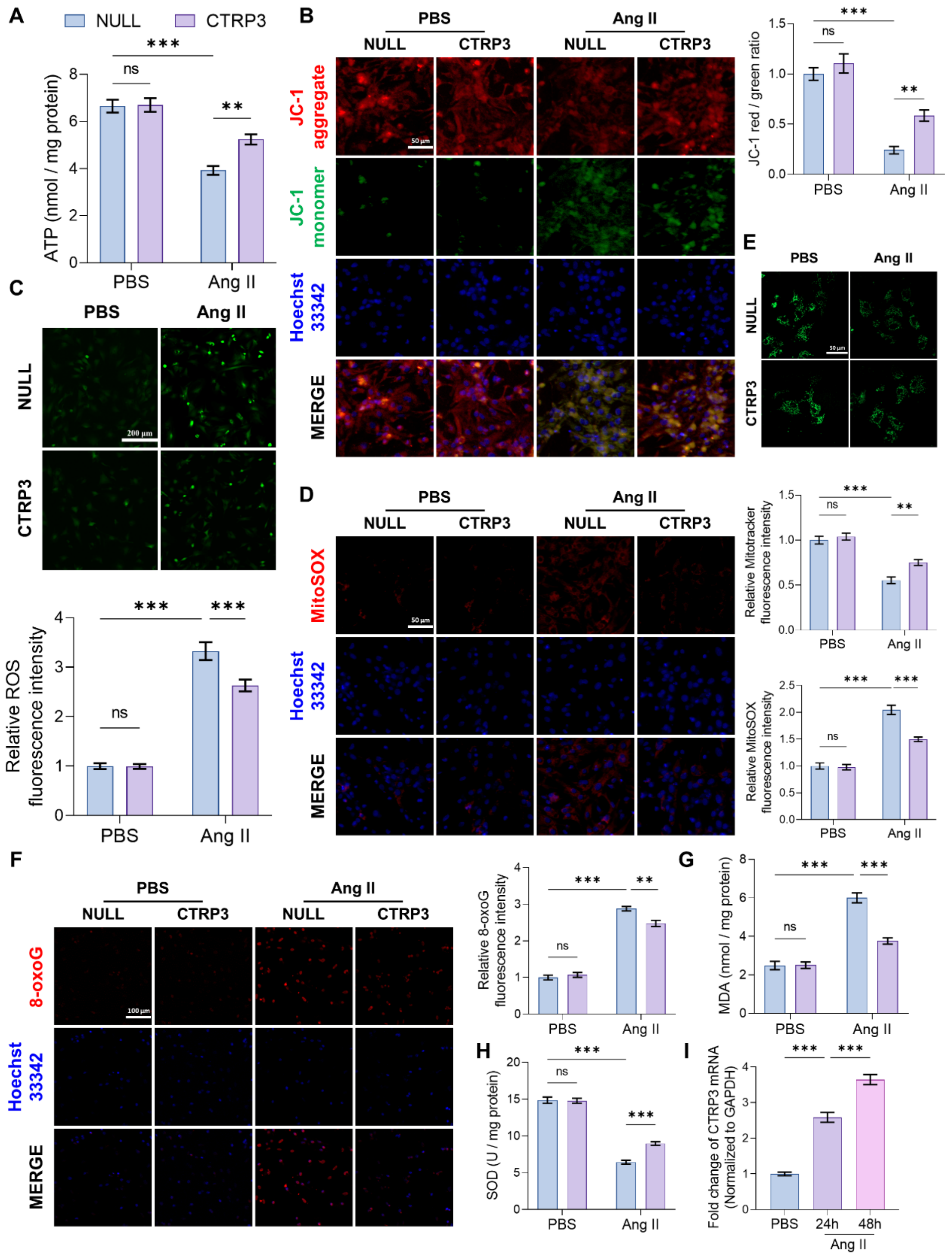

Fig. S2 CTRP3 overexpression alleviates mitochondrial dysfunction and oxidative stress in NRCMs. A Quantification of ATP levels in NRCMs (n = 6). **B** Left panel, representative fluorescence images of mitochondrial transmembrane potential stained with JC-1 in NRCMs. Right panel, relative quantification of the ratio of JC-1 red/green fluorescence intensity in NRCMs (n = 6 samples per group, 10 to 15 random fields were analyzed per sample). **C** Top panel, representative fluorescence images of NRCMs stained with DCFH-DA (green) for indication of ROS level. Bottom panel, relative

quantification of ROS fluorescence intensity of NRCMs (n = 12 samples per group, 10 to 15 random fields were analyzed per sample). **D** Left panel, representative fluorescence images of NRCMs stained with MitoSOX Red (red) to indicate mitochondrial ROS levels. Right panel, relative quantification of mitochondrial ROS fluorescence intensity of NRCMs (n = 6, 10–15 random fields were analyzed per sample). **E** Top panel, representative fluorescence images of mitochondrion stained with MitoTracker™ Green (green) in NRCMs. Bottom panel, relative quantification of MitoTracker™ Green fluorescence intensity of mitochondrion in NRCMs (n = 10 samples per group, 10 to 15 random fields were analyzed per sample). **F** Left panel, representative fluorescence images of NRCMs stained with 8-oxoG (red). Right panel, relative quantification of 8-oxoG fluorescence intensity of NRCMs (n = 6, 10–15 random fields were analyzed per sample). **G** Quantification of MDA levels in NRCMs (n = 9). **H** Quantification of SOD activities in NRCMs (n = 9). **I** Quantification of CTRP3 mRNA levels in NRCMs treated with PBS or Ang II. GAPDH served as the loading control (n = 5). Data were analyzed by one-way ANOVA and presented as mean ± SEM. **p < 0.01; ***p < 0.001; ns, not significant.

Fig. S3

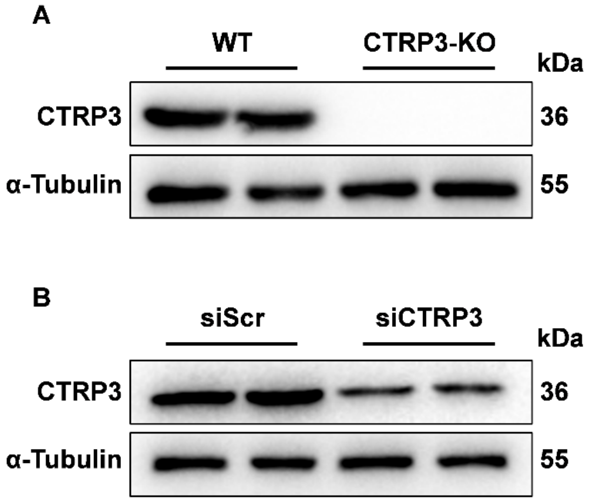

Fig. S3 Effect of CTRP3 intervention in vitro and in vivo. A Representative western blotting analysis of CTRP3 levels in the heart tissues of CTRP3-KO mice. α-Tubulin was used as the loading control. **B** Representative western blotting analysis of CTRP3 levels in NRCMs transfected with siCTRP3. α-Tubulin was used as the loading control.

Fig. S4

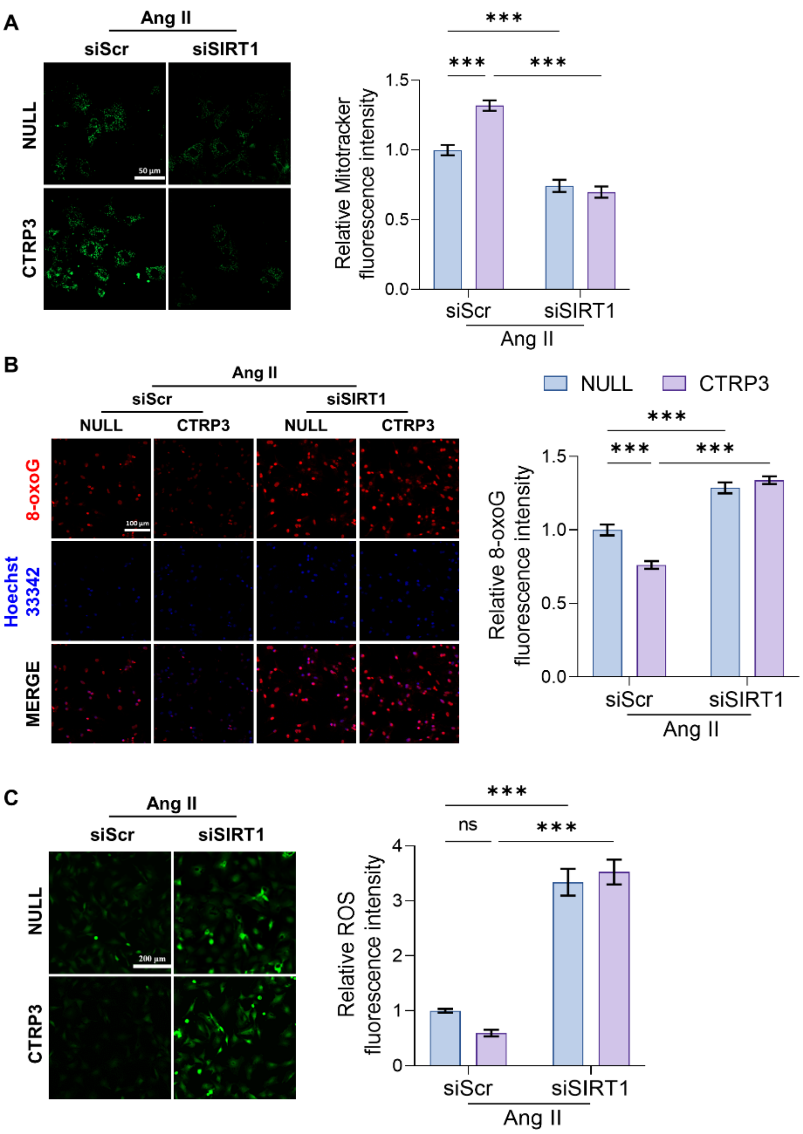

Fig. S4 SIRT1 knockdown prevents the protective effect of CTRP3 against Ang II-induced oxidative stress injury and further aggravates the injury. A Left panel, representative fluorescence images of mitochondria stained with MitoTracker Green (green) in NRCMs. Right panel, relative quantification of MitoTracker™ Green fluorescence intensity of mitochondria in NRCMs (n = 10, 10–15 random fields were analyzed per sample). **B** Left panel, representative fluorescence images of NRCMs stained with 8-oxoG (red). Right panel, relative quantification of 8-oxoG fluorescence intensity of NRCMs (n = 6, 10–15 random fields were analyzed per sample). **C** Left panel, representative fluorescence images of NRCMs stained with DCFH-DA (green) to indicate ROS levels. Right panel, relative quantification of ROS fluorescence intensity of NRCMs (n = 12, 10–15 random fields were analyzed per sample). Data were analyzed by one-way ANOVA and presented as mean ± SEM. ***P < 0.001; ns, not significant.

Table 1

Table 1. Echocardiographic parameters of the left ventricular structure and systolic function in mice (Mean±SD)

	n	IVS;d(mm)	IVS;s(mm)	LVPW;d(mm)	LVPW;s(mm)	LVEF(%)	LVFS(%)
**CTRP3**							
WT	10	0.82±0.04	1.23±0.06	0.80±0.03	1.35±0.06	71.75±2.21	42.04±2.82
KO	10	0.81±0.04	1.21±0.07	0.78±0.04	1.33±0.08	72.30±1.79	41.76±2.52
p-Value		0.60	0.54	0.30	0.55	0.55	0.82
**ATF5**							
WT	10	0.82±0.03	1.33±0.07	0.64±0.03	1.07±0.08	72.31±2.06	35.50±1.60
KO	10	0.80±0.04	1.30±0.06	0.63±0.04	1.09±0.10	71.14±2.13	34.45±1.85
p-Value		0.37	0.29	0.37	0.60	0.23	0.19
**SIRT1**							
WT	10	1.01±0.04	1.42±0.08	1.05±0.05	1.29±0.04	71.30±2.31	33.30±2.08
KO	10	1.00±0.06	1.40±0.08	1.04±0.06	1.24±0.10	69.39±2.73	32.76±2.29
p-Value		0.58	0.50	0.76	0.23	0.11	0.58

IVS;d: Interventricular septum end-diastolic thickness; IVS;s: Interventricular septum end-systolic thickness; LVPW;d: Left ventricular end-diastolic posterior wall thickness; LVPW;s: Left ventricular end-systolic posterior wall thickness; LVEF: Left ventricular ejection fraction; LVFS: Left ventricular fractional shortening.
