# Supplementary material for: CTRP3 alleviates mitochondrial dysfunction and oxidative stress injury in pathological cardiac hypertrophy by activating UPRmt via the SIRT1/ATF5 axis
Source: Cell Death Discov. 2024 Jan 26;10:53. doi: 10.1038/s41420-024-01813-x (PMC10817931; doi:10.1038/s41420-024-01813-x)

Figure 1B

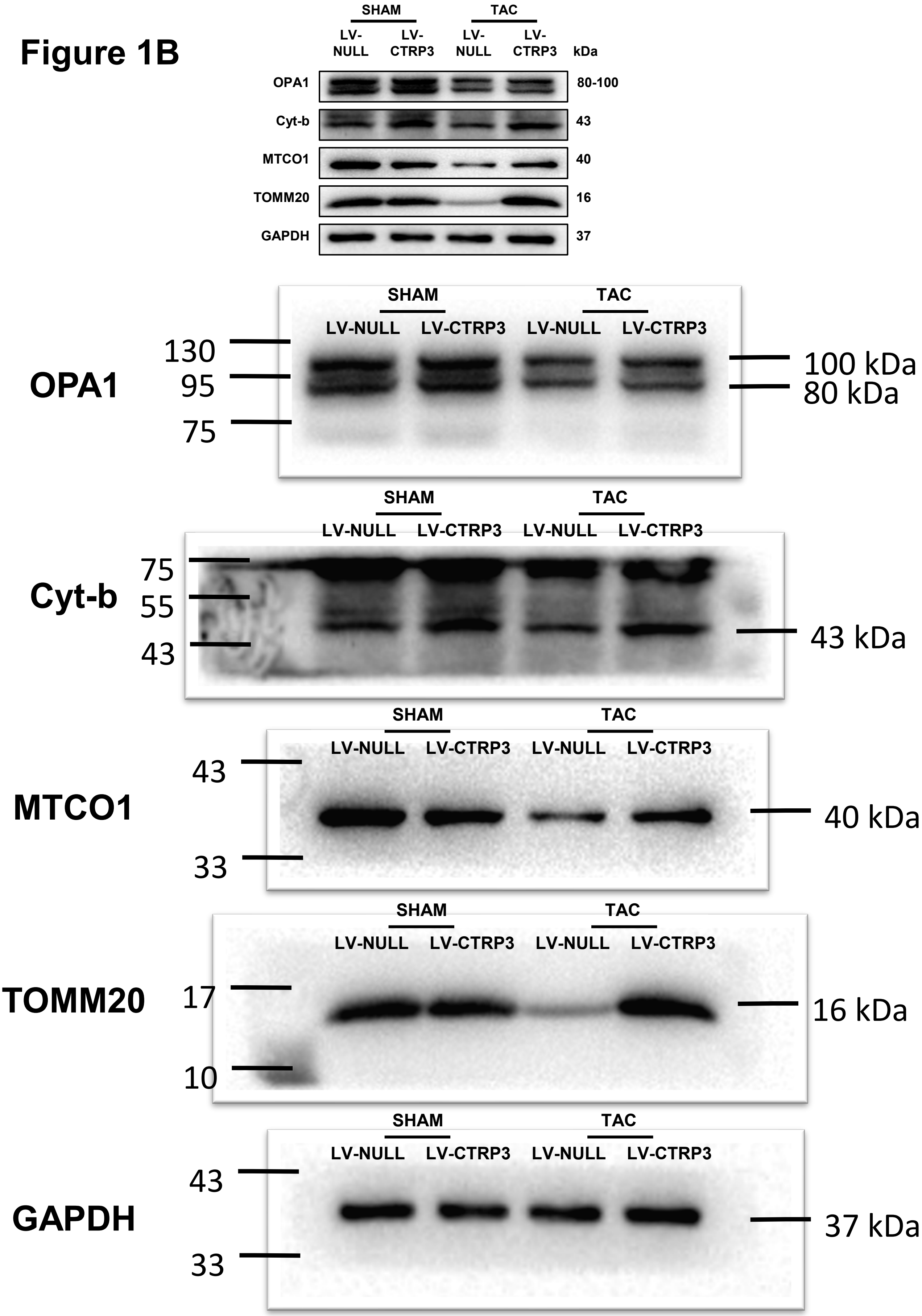
SHAM
TAC
LV-NULL
LV-CTRP3
LV-NULL
LV-CTRP3
kDa
OPA1
80-100
Cyt-b
43
MTCO1
40
TOMM20
16
GAPDH
37
OPA1
130
95
75
100 kDa
80 kDa
Cyt-b
75
55
43
43 kDa
MTCO1
43
33
40 kDa
TOMM20
17
10
16 kDa
GAPDH
43
33
37 kDa

Figure 2A

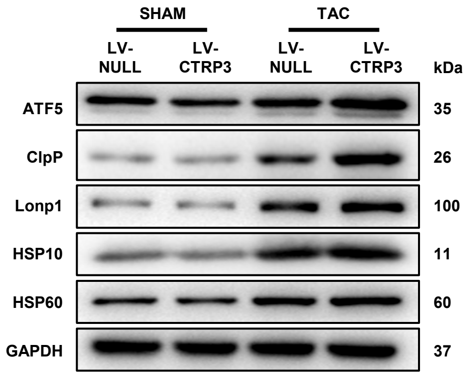
SHAM
TAC
LV-NULL
LV-CTRP3
LV-NULL
LV-CTRP3
kDa
ATF5
35
ClpP
26
Lonp1
100
HSP10
11
HSP60
60
GAPDH
37

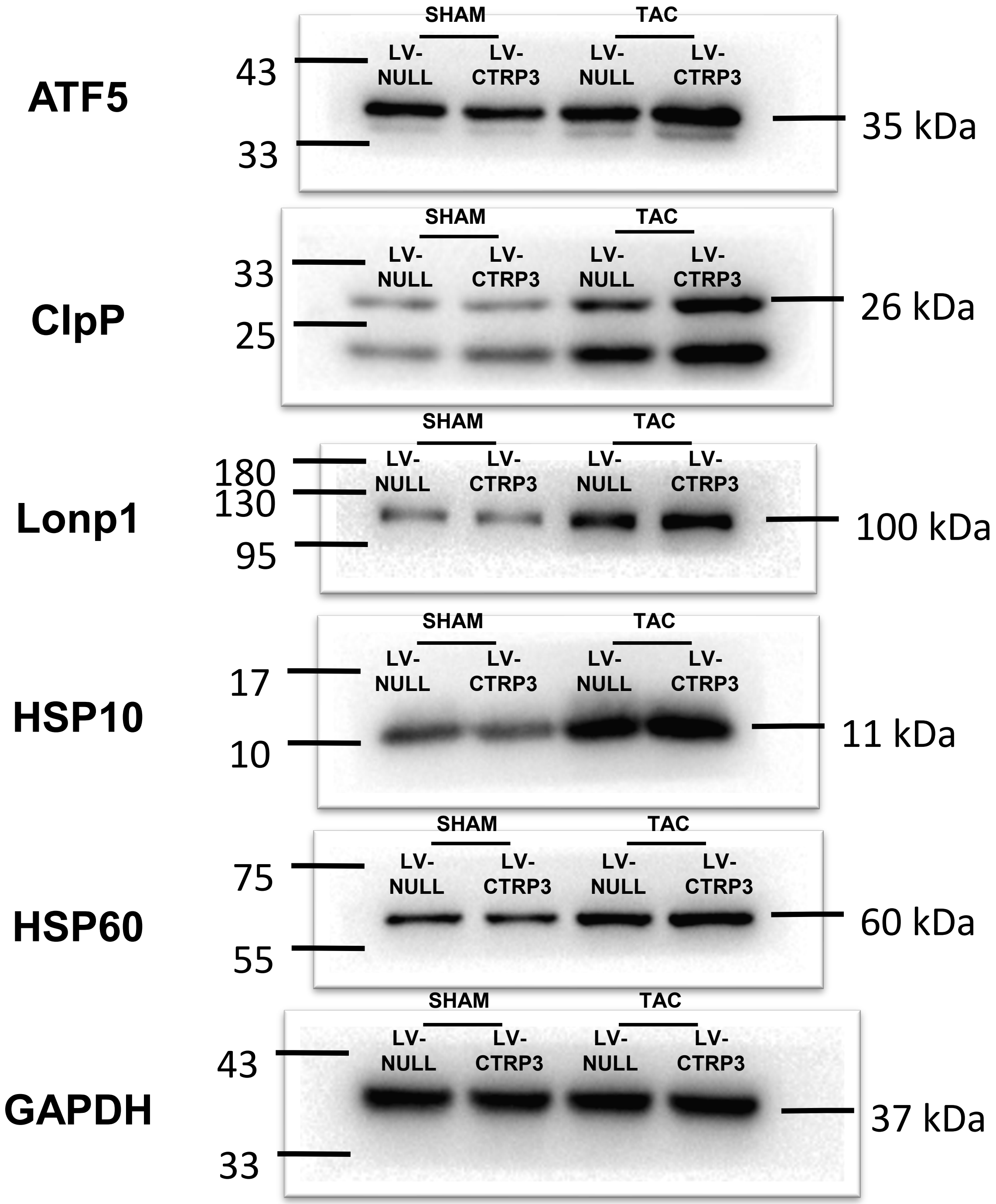
ATF5
SHAM
TAC
LV-NULL
LV-CTRP3
LV-NULL
LV-CTRP3
43
33
35 kDa
ClpP
33
25
26 kDa
Lonp1
180
130
95
100 kDa
HSP10
17
10
11 kDa
HSP60
75
55
60 kDa
GAPDH
43
33
37 kDa

Figure 2B

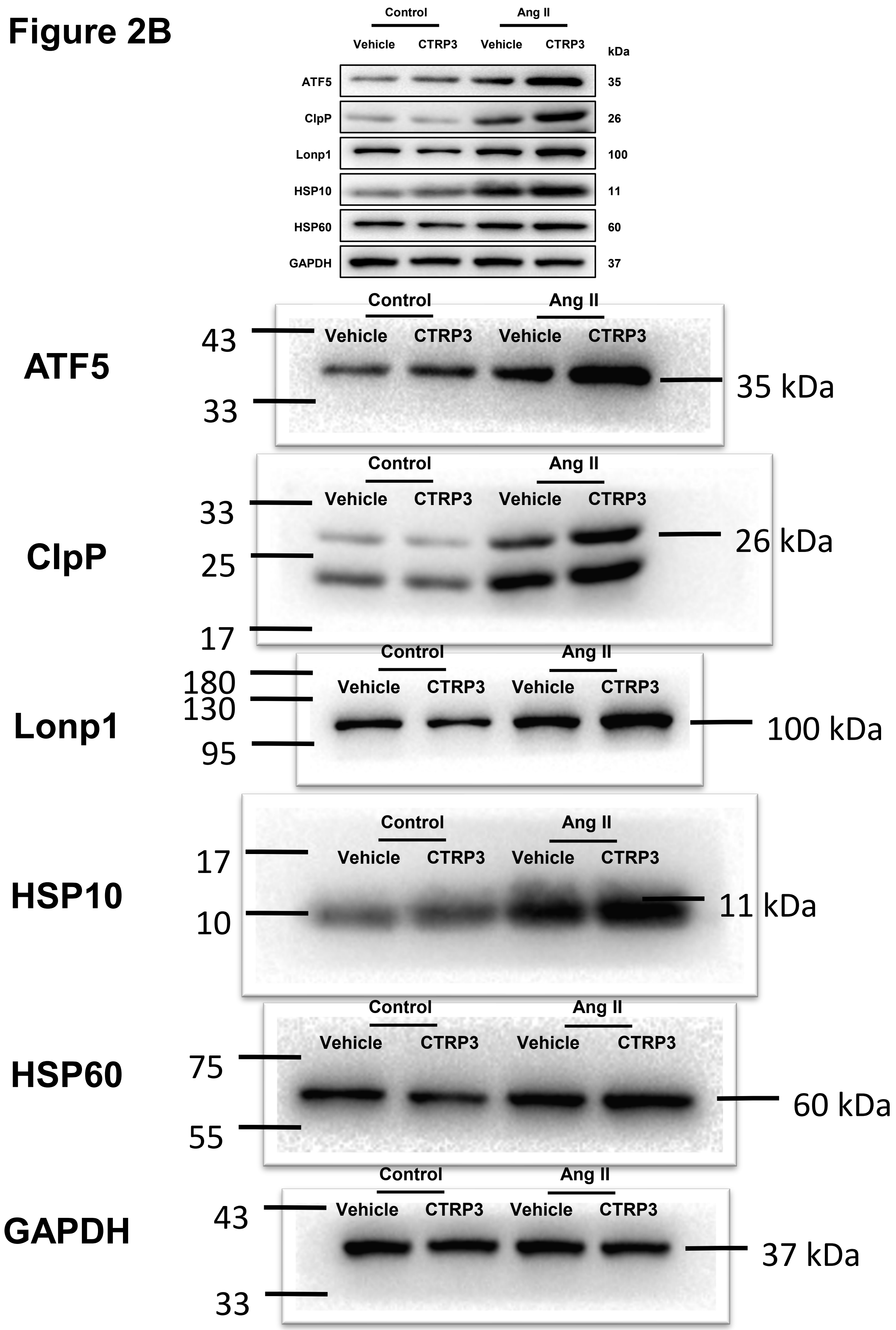

Control
Ang II
Vehicle
CTRP3
Vehicle
CTRP3
kDa
ATF5 35
ClpP 26
Lonp1 100
HSP10 11
HSP60 60
GAPDH 37
ATF5
43
33
35 kDa
ClpP
33
25
17
26 kDa
Lonp1
180
130
95
100 kDa
HSP10
17
10
11 kDa
HSP60
75
55
60 kDa
GAPDH
43
33
37 kDa

Figure 2C

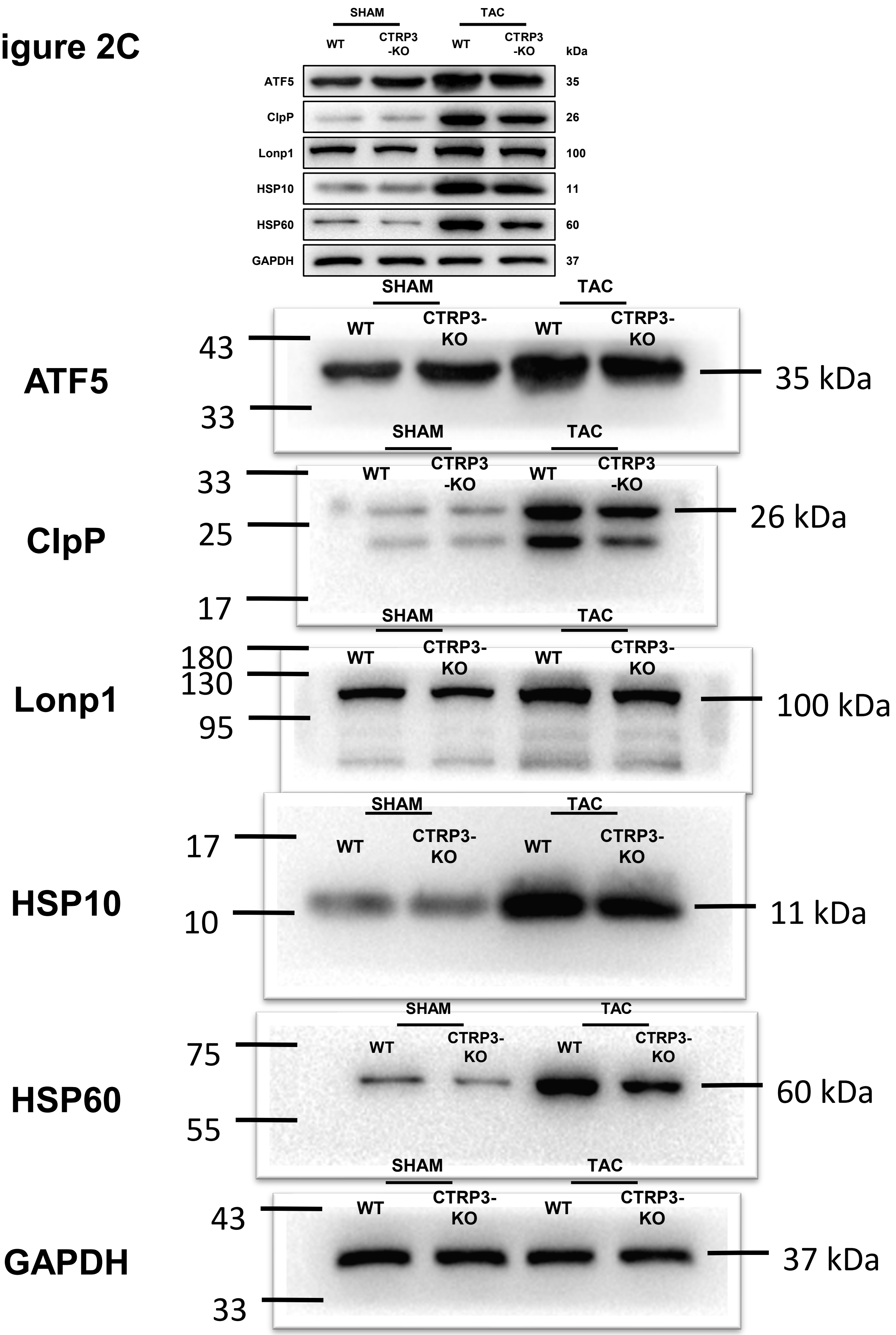
SHAM
TAC
WT
CTRP3 -KO
kDa
ATF5
35
ClpP
26
Lonp1
100
HSP10
11
HSP60
60
GAPDH
37
ATF5
43
33
35 kDa
ClpP
33
25
17
26 kDa
Lonp1
180
130
95
100 kDa
HSP10
17
10
11 kDa
HSP60
75
55
60 kDa
GAPDH
43
33
37 kDa

Figure 2D

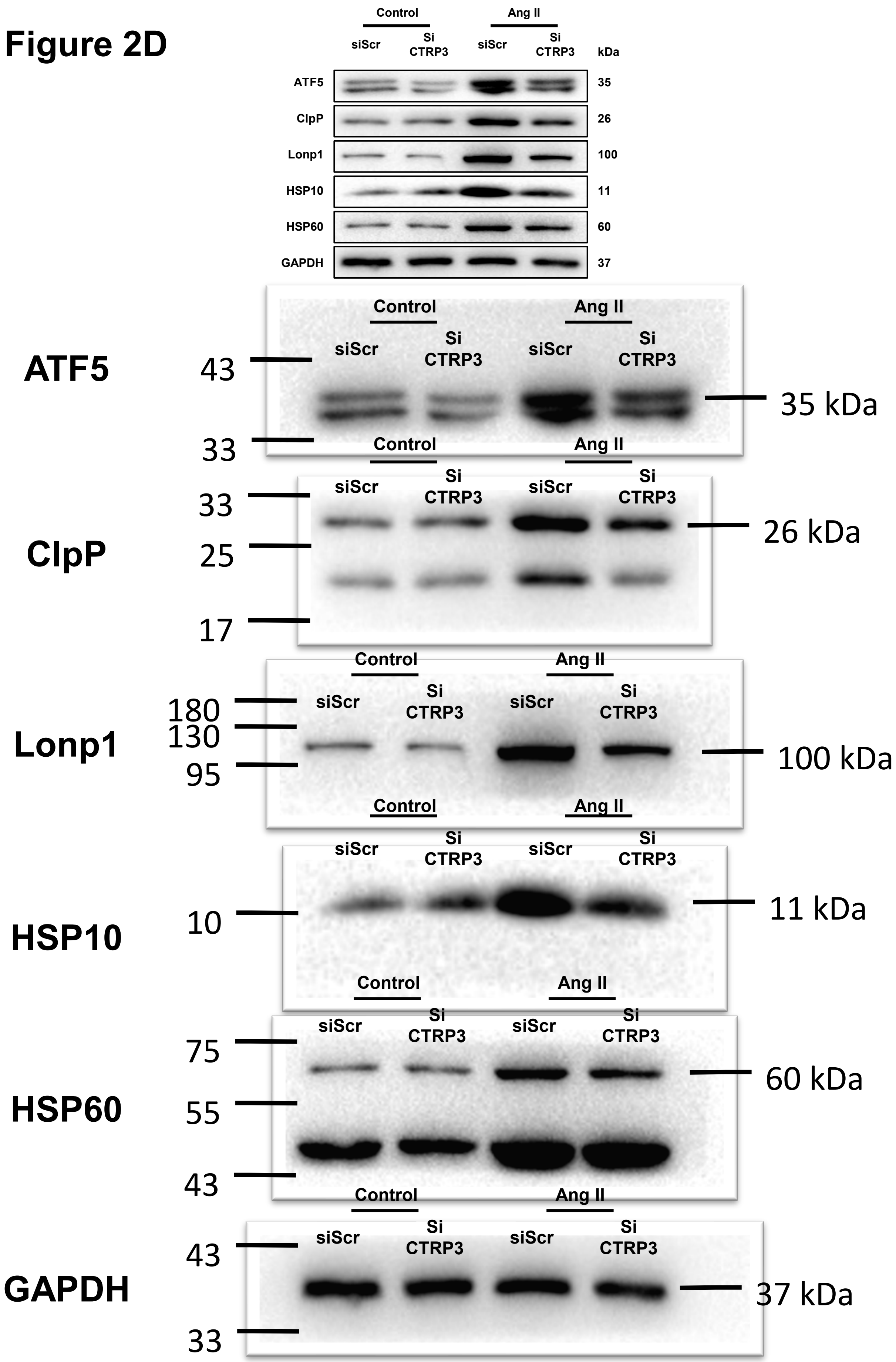
Control
Ang II
siScr
Si CTRP3
kDa
ATF5 35
ClpP 26
Lonp1 100
HSP10 11
HSP60 60
GAPDH 37
ATF5
43
33
35 kDa
ClpP
33
25
17
26 kDa
Lonp1
180
130
95
100 kDa
HSP10
10
11 kDa
HSP60
75
55
43
60 kDa
GAPDH
43
33
37 kDa

Figure 3A

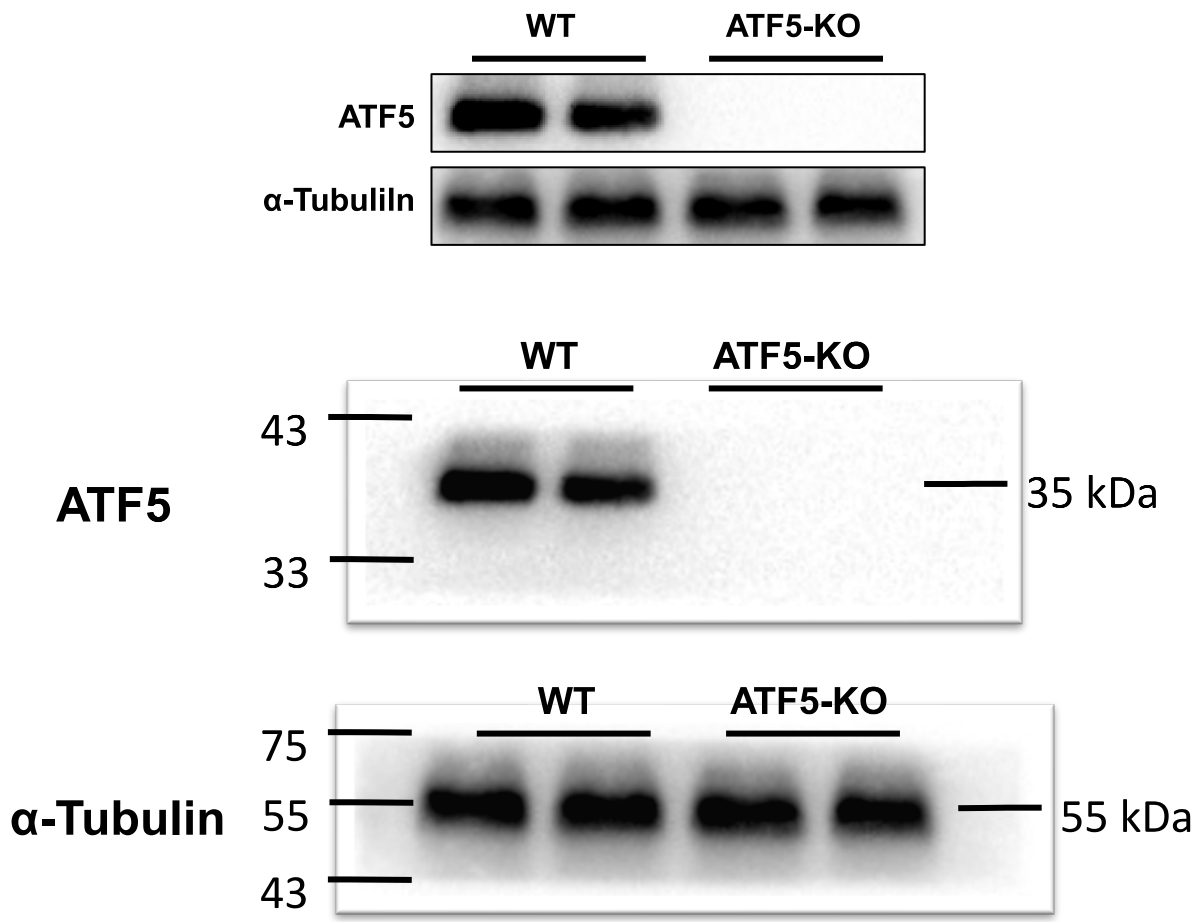

WT
ATF5-KO
ATF5
α-Tubuliln
WT
ATF5-KO
43
ATF5
35 kDa
33
WT
ATF5-KO
75
α-Tubulin
55
55 kDa
43

Figure 3D

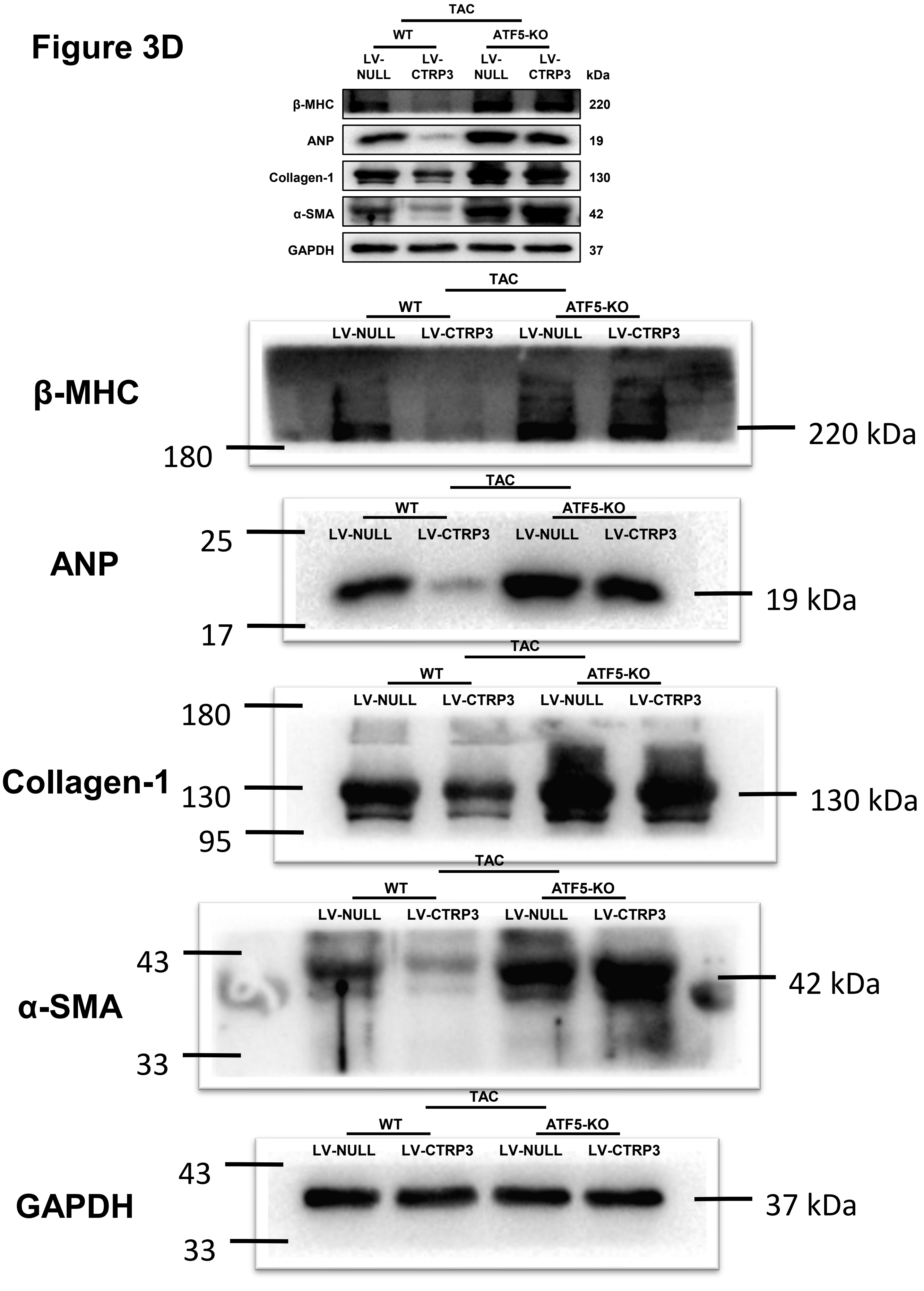
TAC
WT
ATF5-KO
LV-NULL
LV-CTRP3
kDa
β-MHC 220
ANP 19
Collagen-1 130
α-SMA 42
GAPDH 37
β-MHC
180
220 kDa
ANP
25
17
19 kDa
Collagen-1
180
130
95
130 kDa
α-SMA
43
33
42 kDa
GAPDH
43
33
37 kDa

Figure 3E

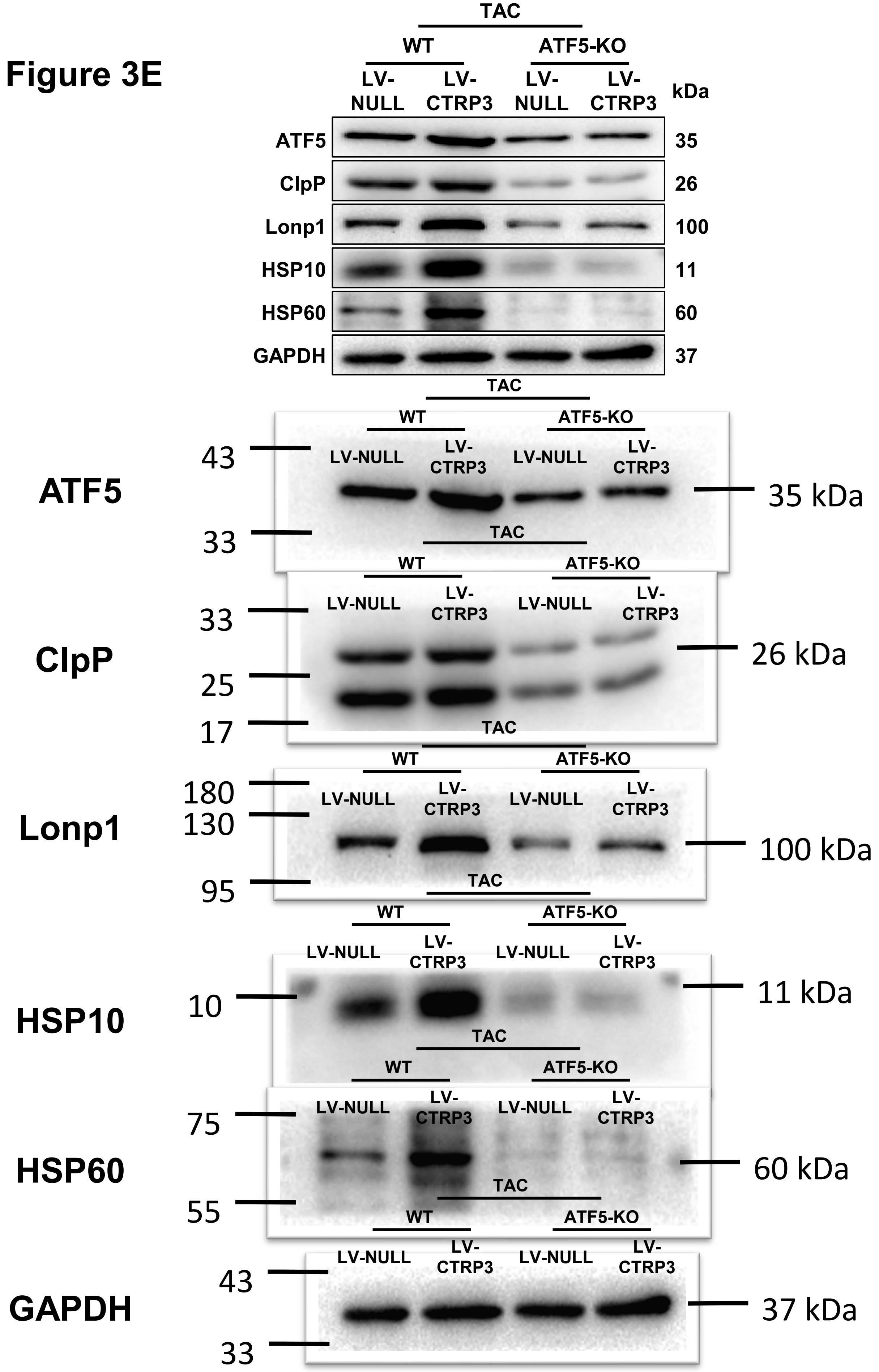
TAC
WT
ATF5-KO
LV-NULL
LV-CTRP3
LV-NULL
LV-CTRP3
kDa
ATF5 35
ClpP 26
Lonp1 100
HSP10 11
HSP60 60
GAPDH 37
ATF5
43
33
35 kDa
ClpP
33
25
17
26 kDa
Lonp1
180
130
95
100 kDa
HSP10
10
11 kDa
HSP60
75
55
60 kDa
GAPDH
43
33
37 kDa

Figure 4A

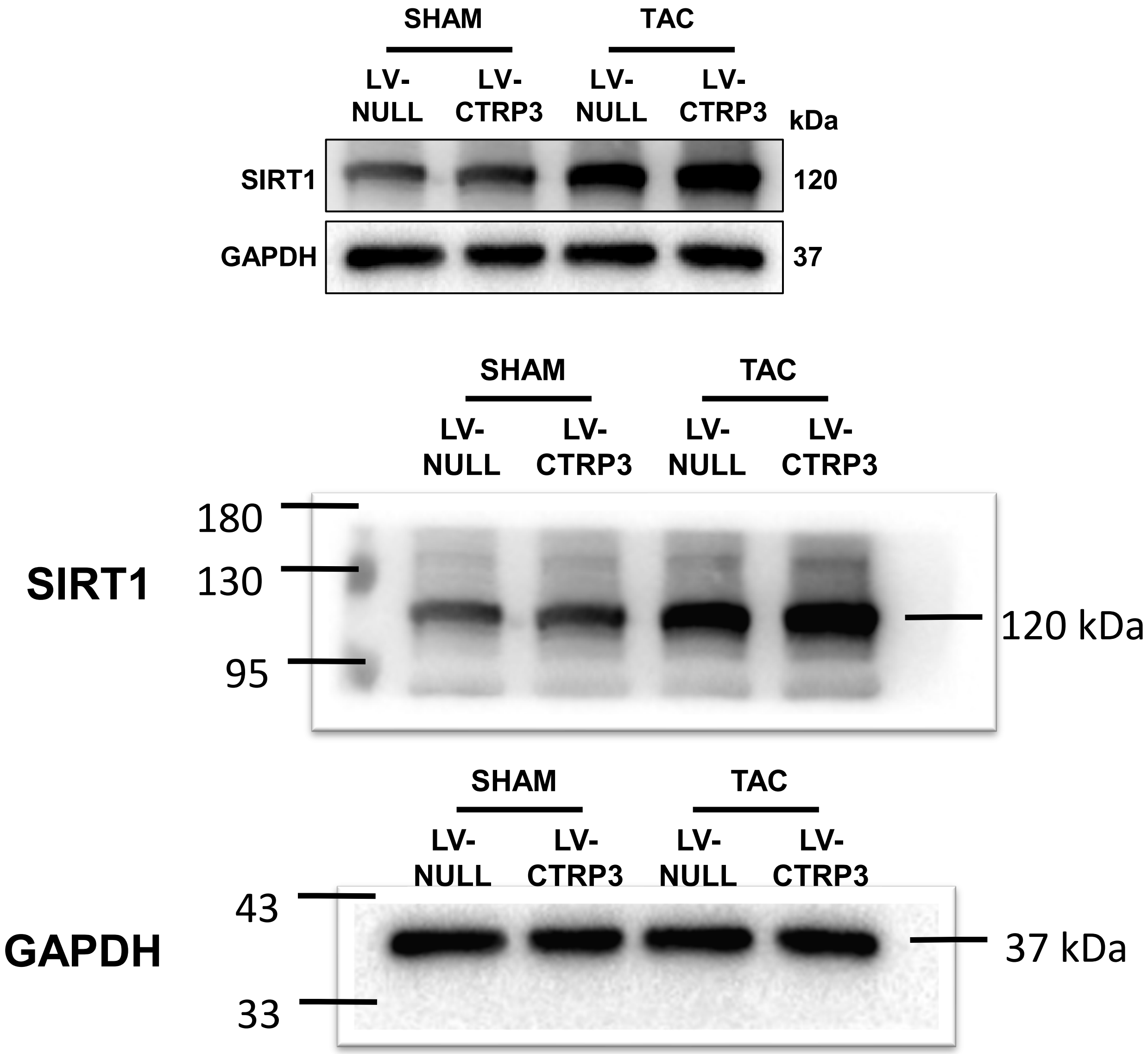
SHAM
TAC
LV-NULL
LV-CTRP3
LV-NULL
LV-CTRP3
kDa
SIRT1
120
GAPDH
37
SHAM
TAC
LV-NULL
LV-CTRP3
LV-NULL
LV-CTRP3
SIRT1
180
130
95
120 kDa
SHAM
TAC
LV-NULL
LV-CTRP3
LV-NULL
LV-CTRP3
GAPDH
43
33
37 kDa

Figure 5A

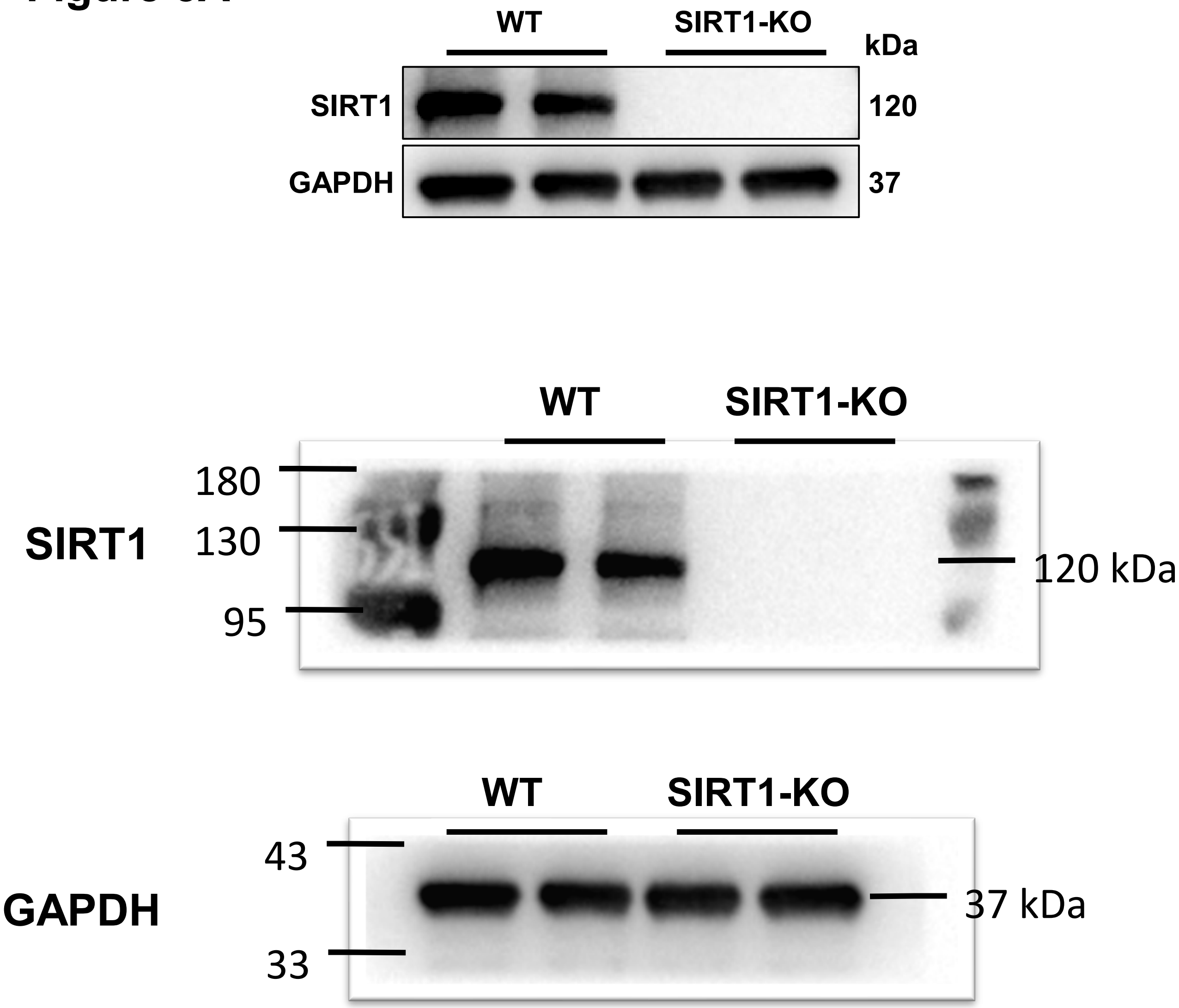
WT
SIRT1-KO
kDa
SIRT1
120
GAPDH
37
WT
SIRT1-KO
180
130
95
SIRT1
120 kDa
WT
SIRT1-KO
43
33
GAPDH
37 kDa

Figure 5D

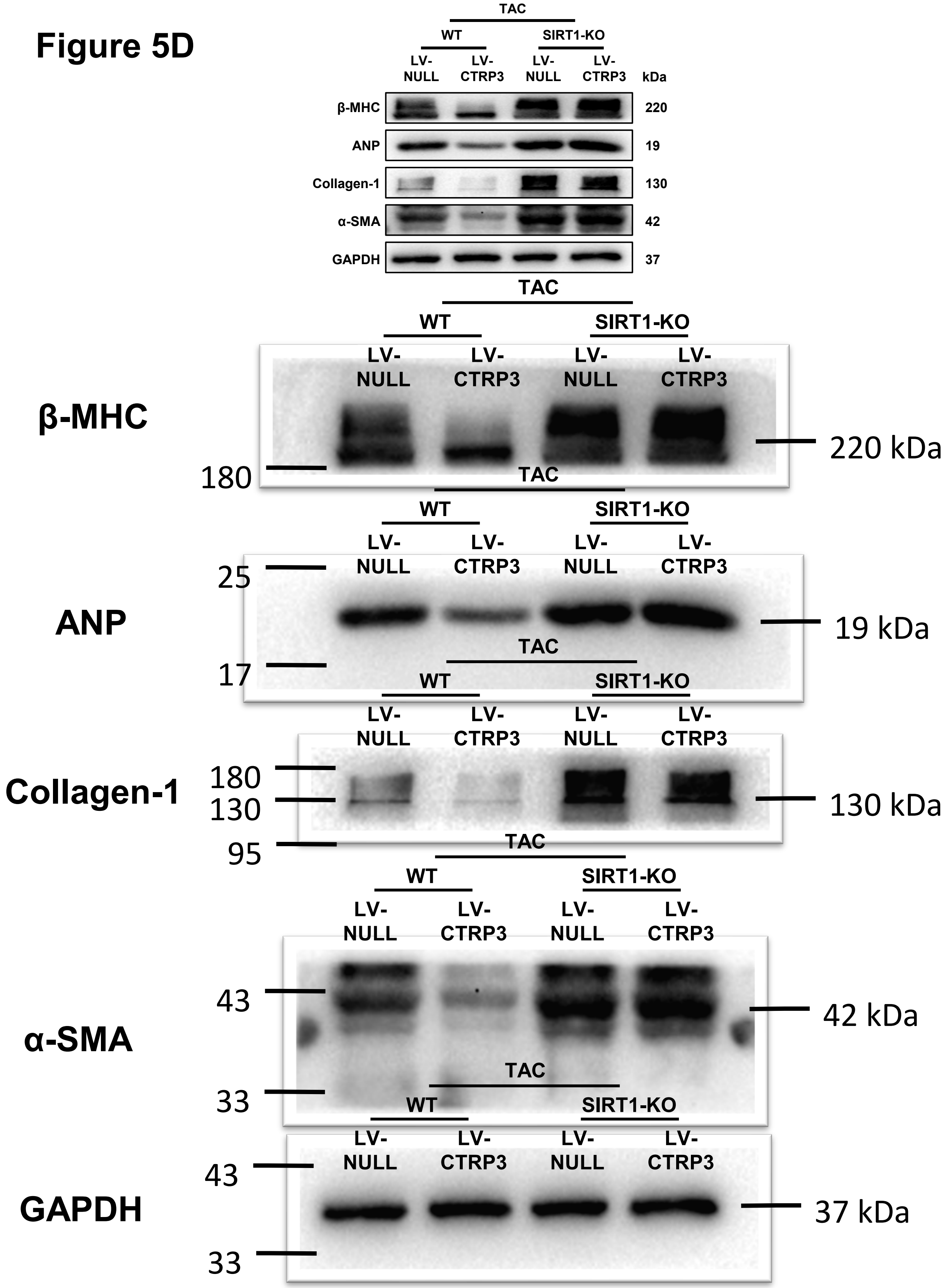
TAC
WT
SIRT1-KO
LV-NULL
LV-CTRP3
LV-NULL
LV-CTRP3
kDa
β-MHC 220
ANP 19
Collagen-1 130
α-SMA 42
GAPDH 37
β-MHC
180
220 kDa
ANP
25
17
19 kDa
Collagen-1
180
130
95
130 kDa
α-SMA
43
33
42 kDa
GAPDH
43
33
37 kDa

Figure 5E

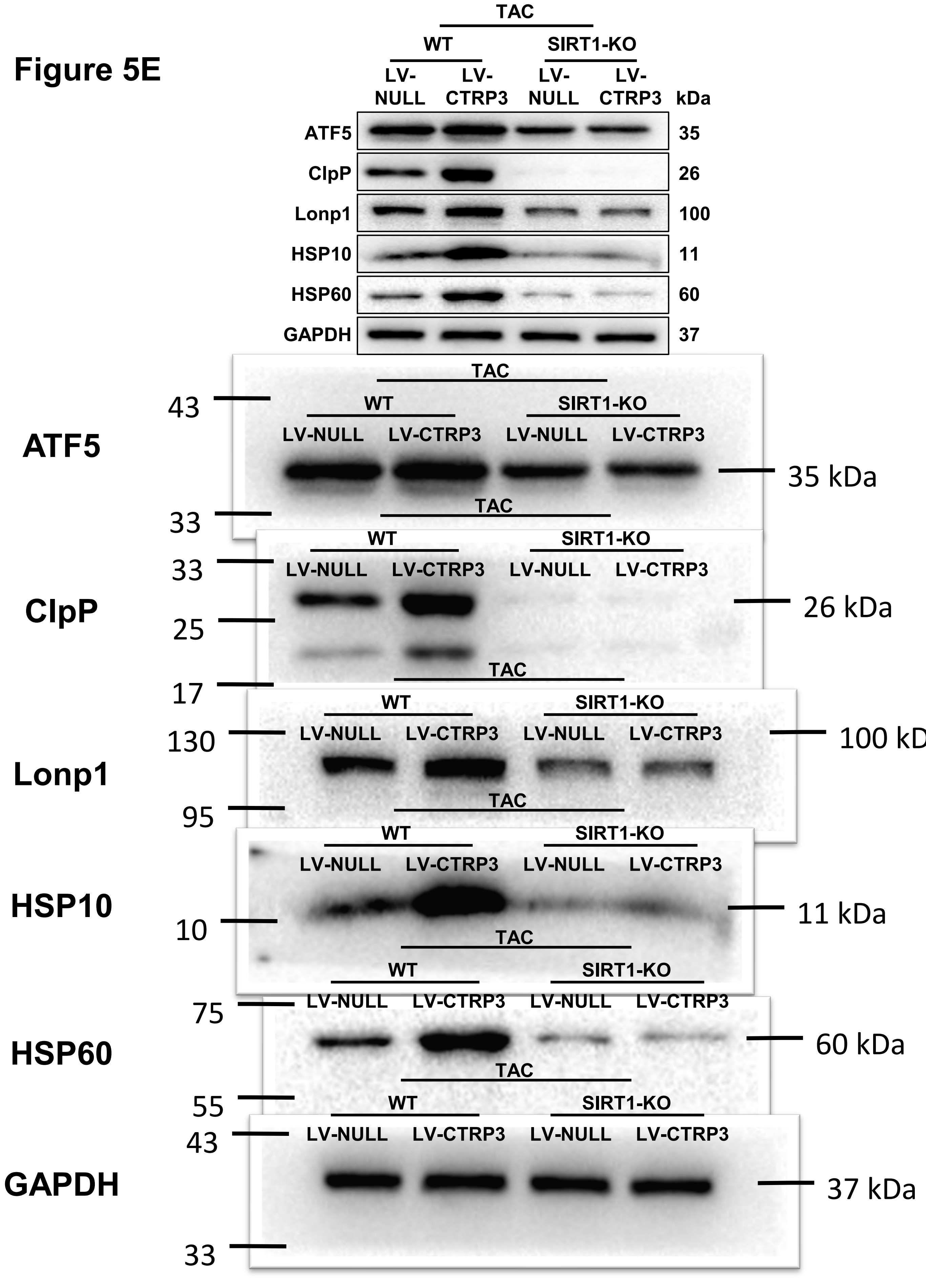
TAC
WT
SIRT1-KO
LV-NULL
LV-CTRP3
kDa
ATF5 35
ClpP 26
Lonp1 100
HSP10 11
HSP60 60
GAPDH 37
ATF5
43
33
35 kDa
ClpP
33
25
17
26 kDa
Lonp1
130
95
100 kD
HSP10
10
11 kDa
HSP60
75
55
60 kDa
GAPDH
43
33
37 kDa

Figure 6A

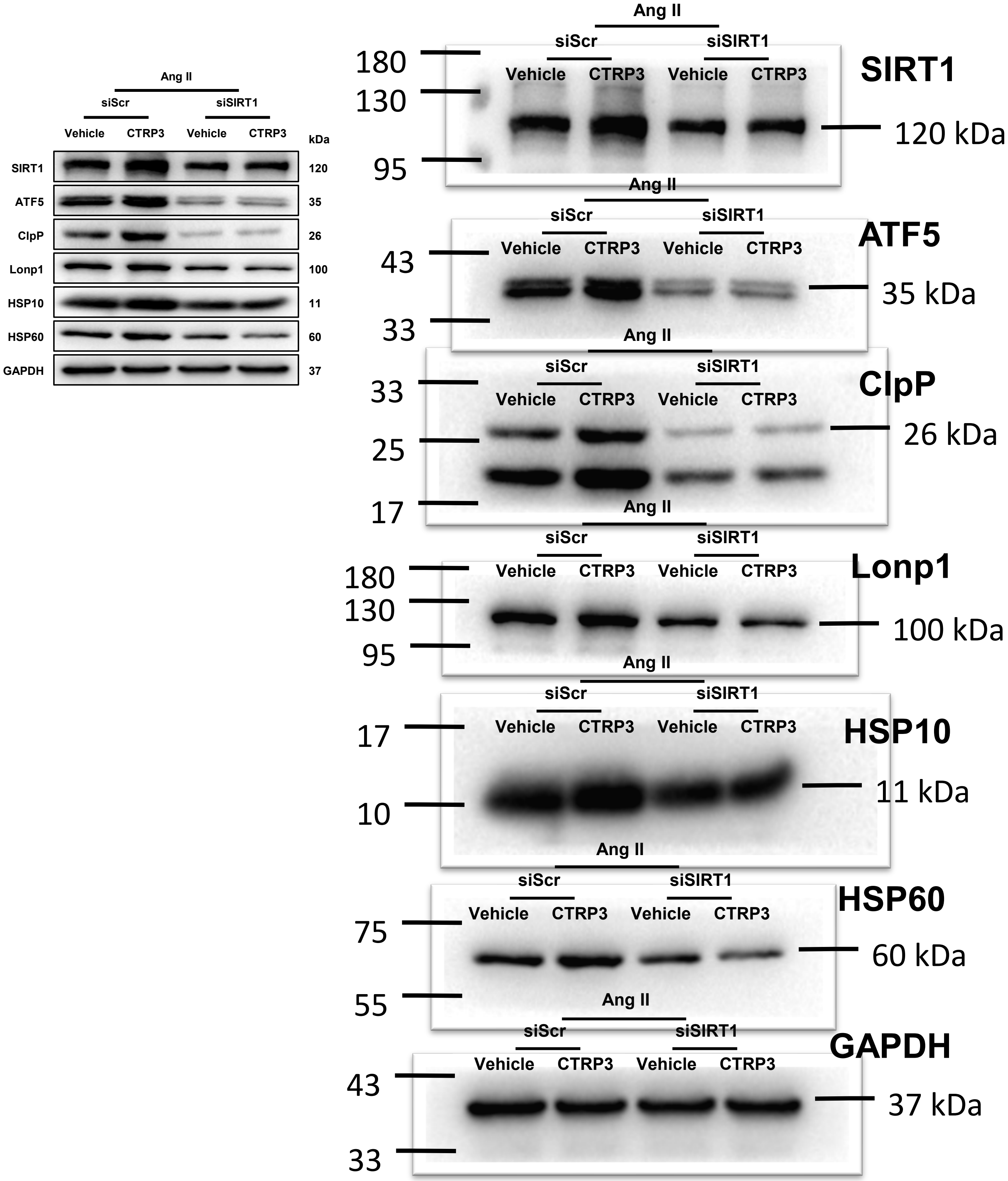

Ang II
siScr
siSIRT1
Vehicle
CTRP3
kDa
SIRT1
ATF5
ClpP
Lonp1
HSP10
HSP60
GAPDH
120
35
26
100
11
60
37
180
130
95
120 kDa
43
33
35 kDa
25
17
26 kDa
100 kDa
10
11 kDa
75
55
60 kDa
37 kDa

Figure 6B

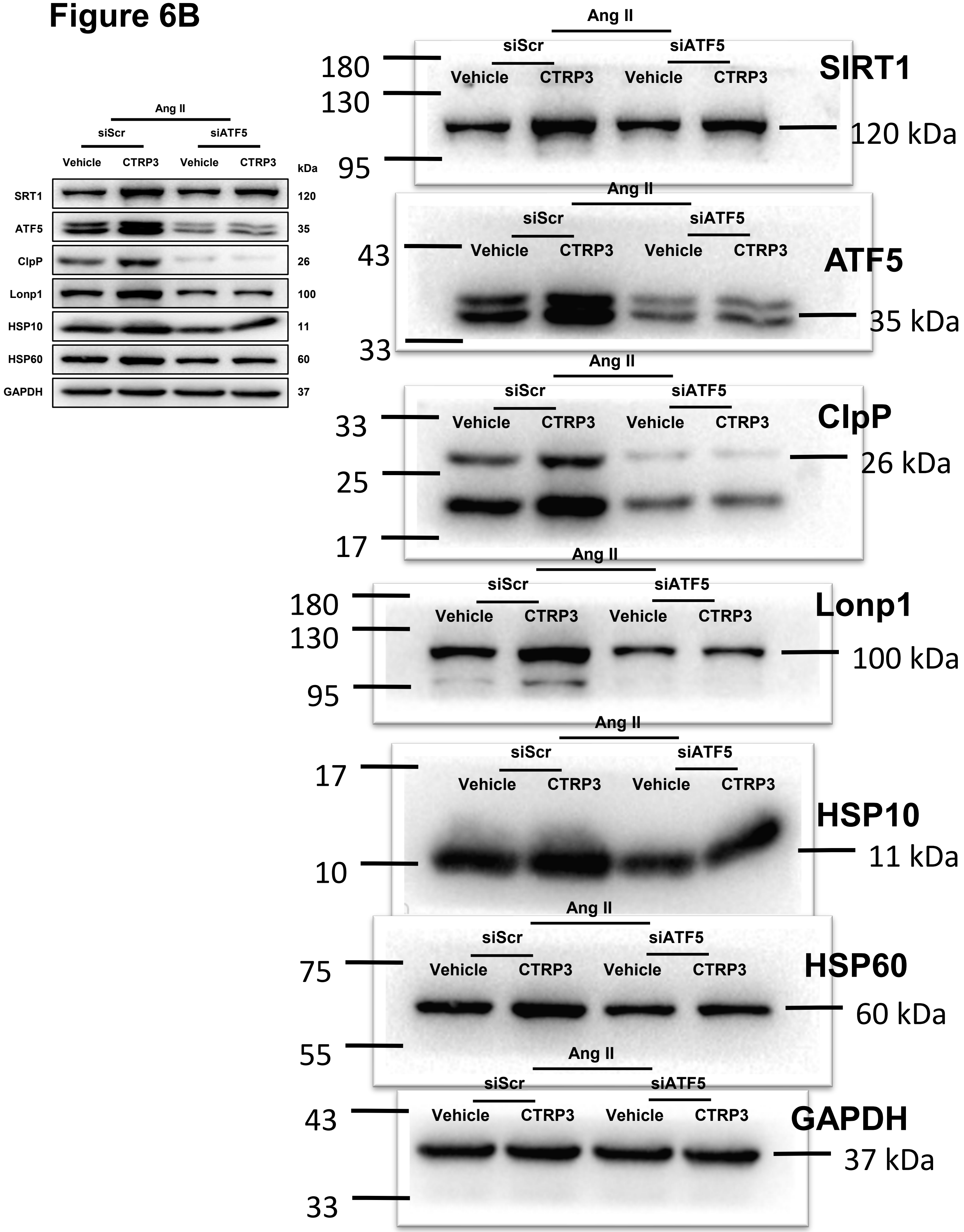

Ang II
siScr
siATF5
Vehicle
CTRP3
Vehicle
CTRP3
kDa
SIRT1 120
ATF5 35
ClpP 26
Lonp1 100
HSP10 11
HSP60 60
GAPDH 37
SIRT1
180
130
95
120 kDa
ATF5
43
33
35 kDa
ClpP
33
25
17
26 kDa
Lonp1
180
130
95
100 kDa
HSP10
17
10
11 kDa
HSP60
75
55
60 kDa
GAPDH
43
33
37 kDa

Figure 6C

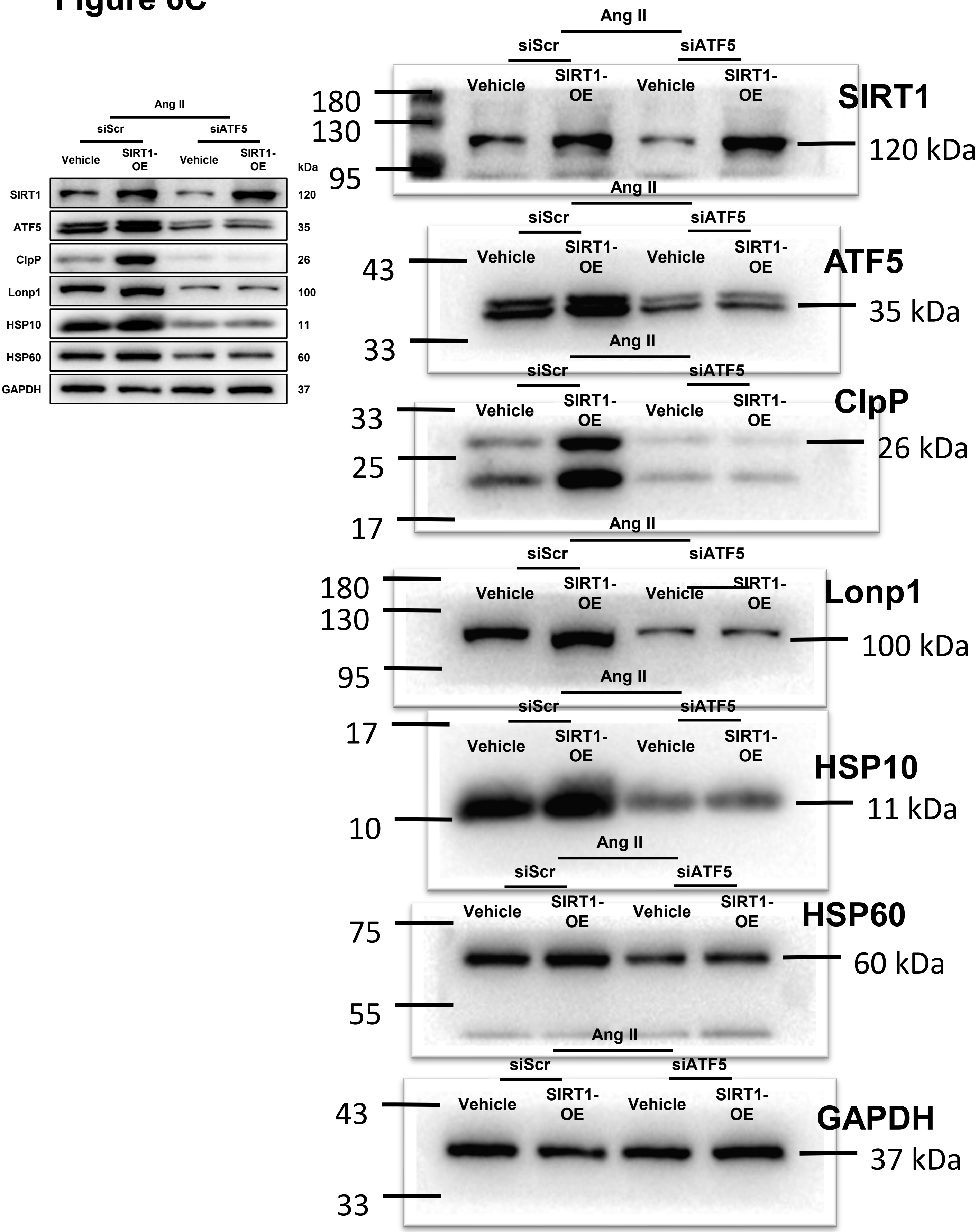

Ang II
siScr
siATF5
Vehicle
SIRT1-OE
Vehicle
SIRT1-OE
kDa
SIRT1 120
ATF5 35
ClpP 26
Lonp1 100
HSP10 11
HSP60 60
GAPDH 37
SIRT1
180
130
95
120 kDa
ATF5
43
33
35 kDa
ClpP
33
25
17
26 kDa
Lonp1
180
130
95
100 kDa
HSP10
17
10
11 kDa
HSP60
75
55
60 kDa
GAPDH
43
33
37 kDa

Figure 6D

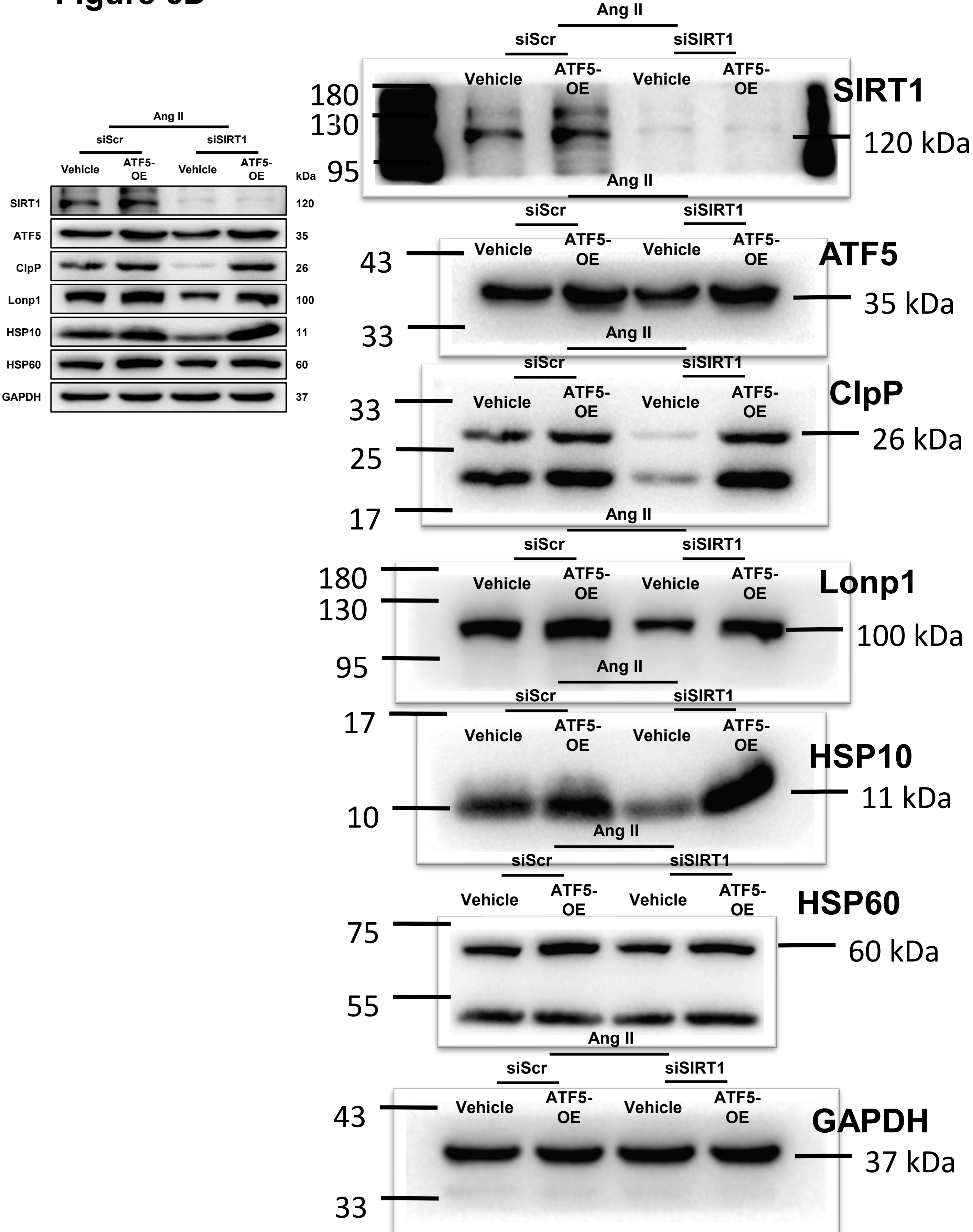

Ang II
siScr
siSIRT1
Vehicle
ATF5-OE
kDa
SIRT1 120
ATF5 35
ClpP 26
Lonp1 100
HSP10 11
HSP60 60
GAPDH 37
SIRT1
180
130
95
120 kDa
ATF5
43
33
35 kDa
ClpP
33
25
17
26 kDa
Lonp1
180
130
95
100 kDa
HSP10
17
10
11 kDa
HSP60
75
55
60 kDa
GAPDH
43
33
37 kDa

Figure S1A

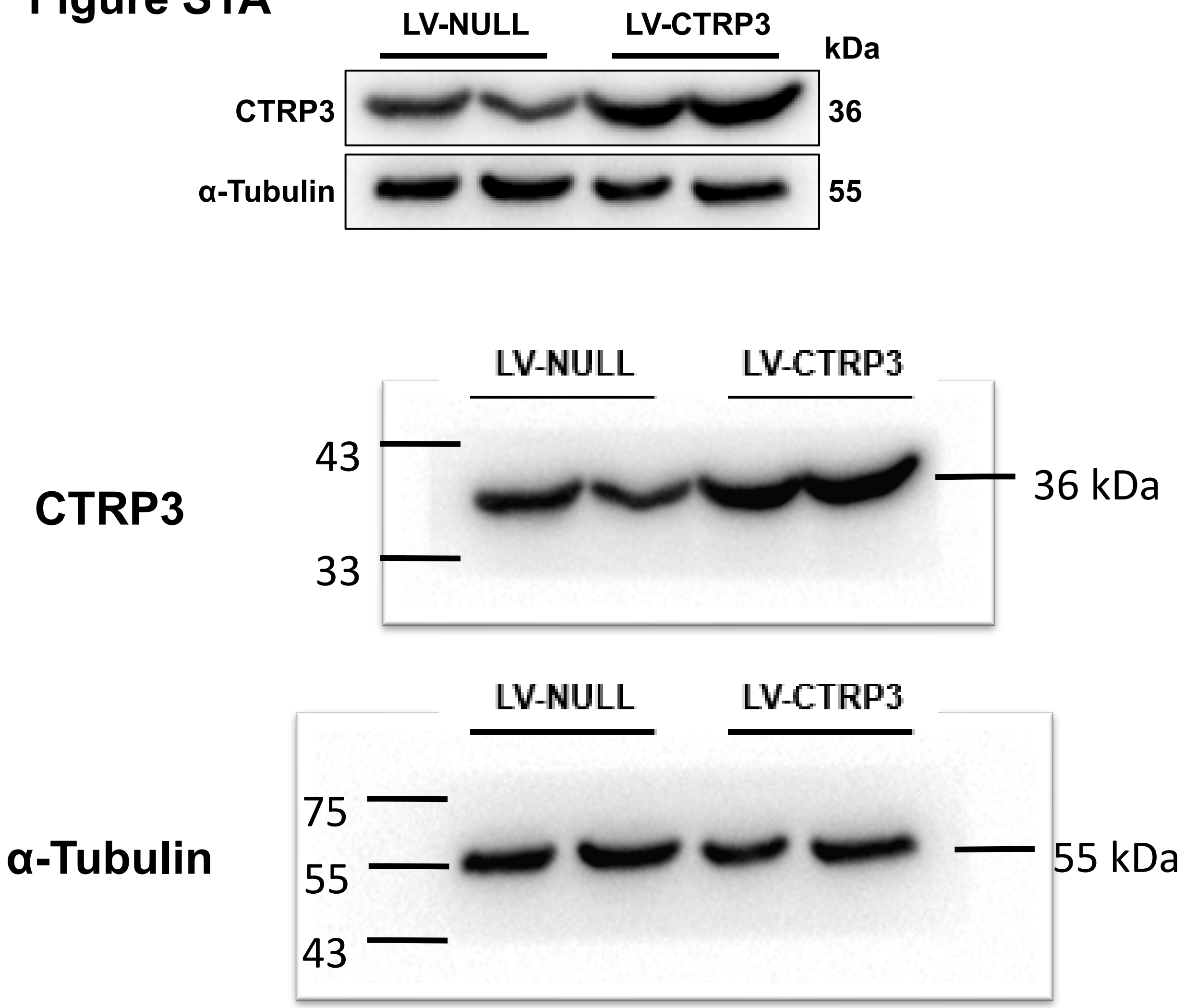
LV-NULL
LV-CTRP3
kDa
CTRP3
36
α-Tubulin
55
LV-NULL
LV-CTRP3
43
CTRP3
36 kDa
33
LV-NULL
LV-CTRP3
75
α-Tubulin
55
55 kDa
43

Figure S1C

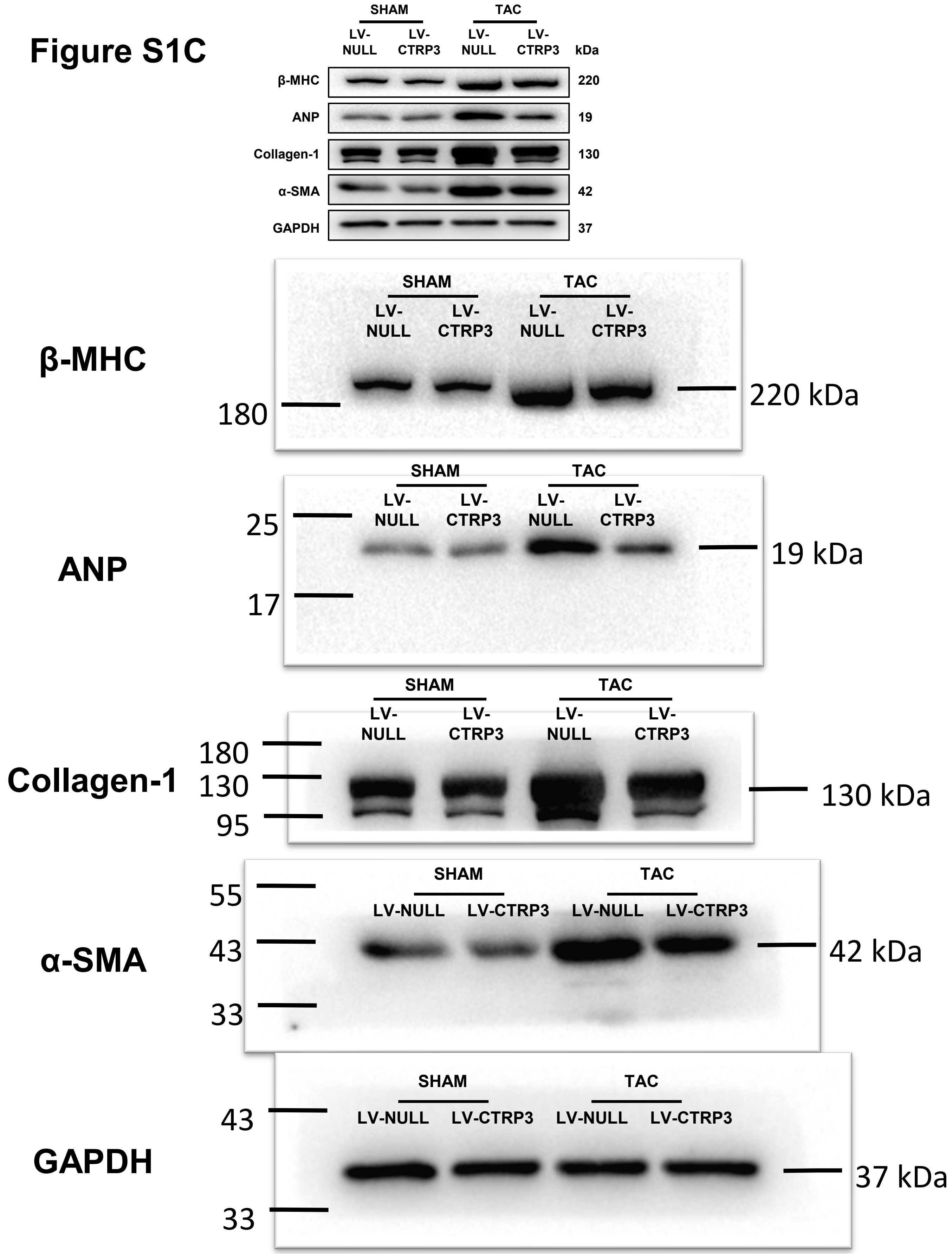
SHAM
TAC
LV-NULL
LV-CTRP3
kDa
β-MHC
ANP
Collagen-1
α-SMA
GAPDH
220
19
130
42
37
180
220 kDa
25
17
19 kDa
95
130 kDa
55
43
33
42 kDa
37 kDa

Figure S3A

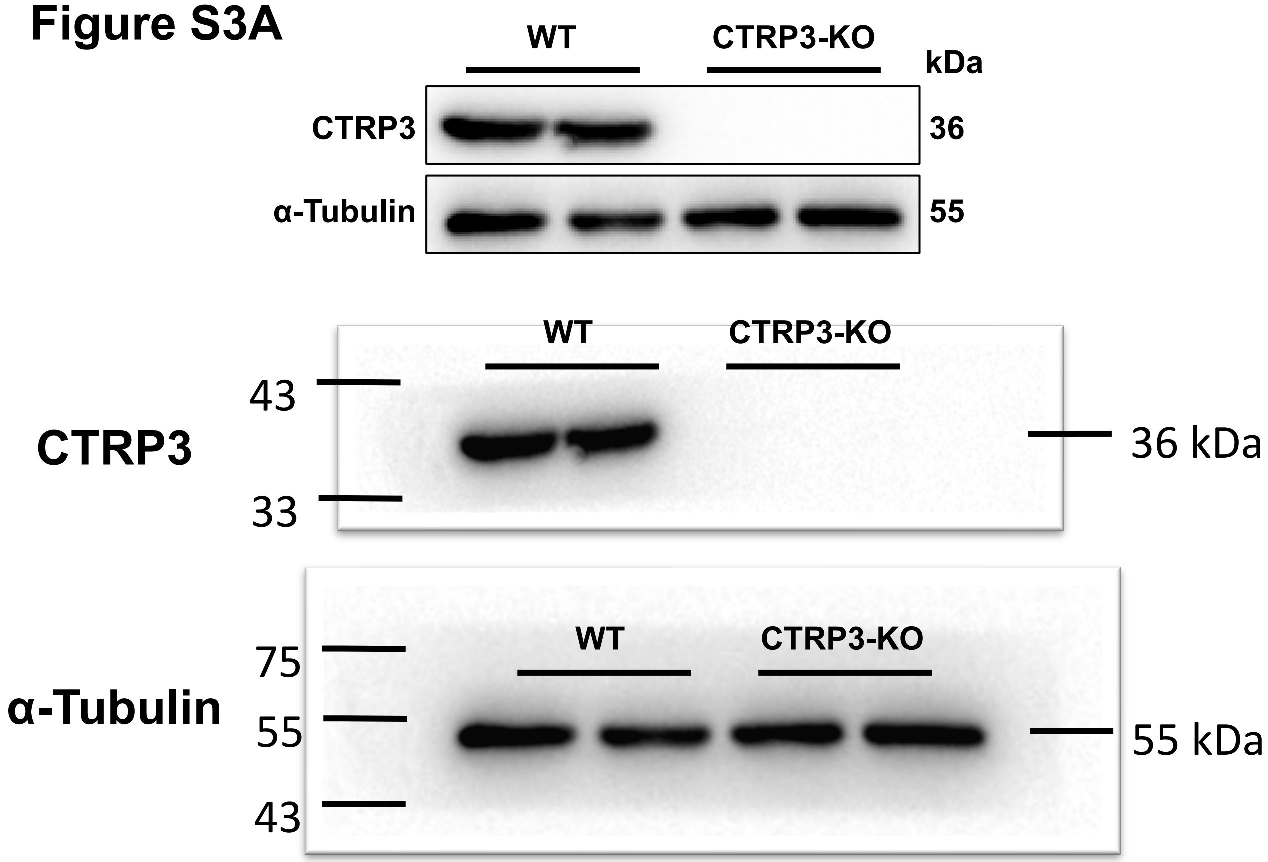

Figure S3B

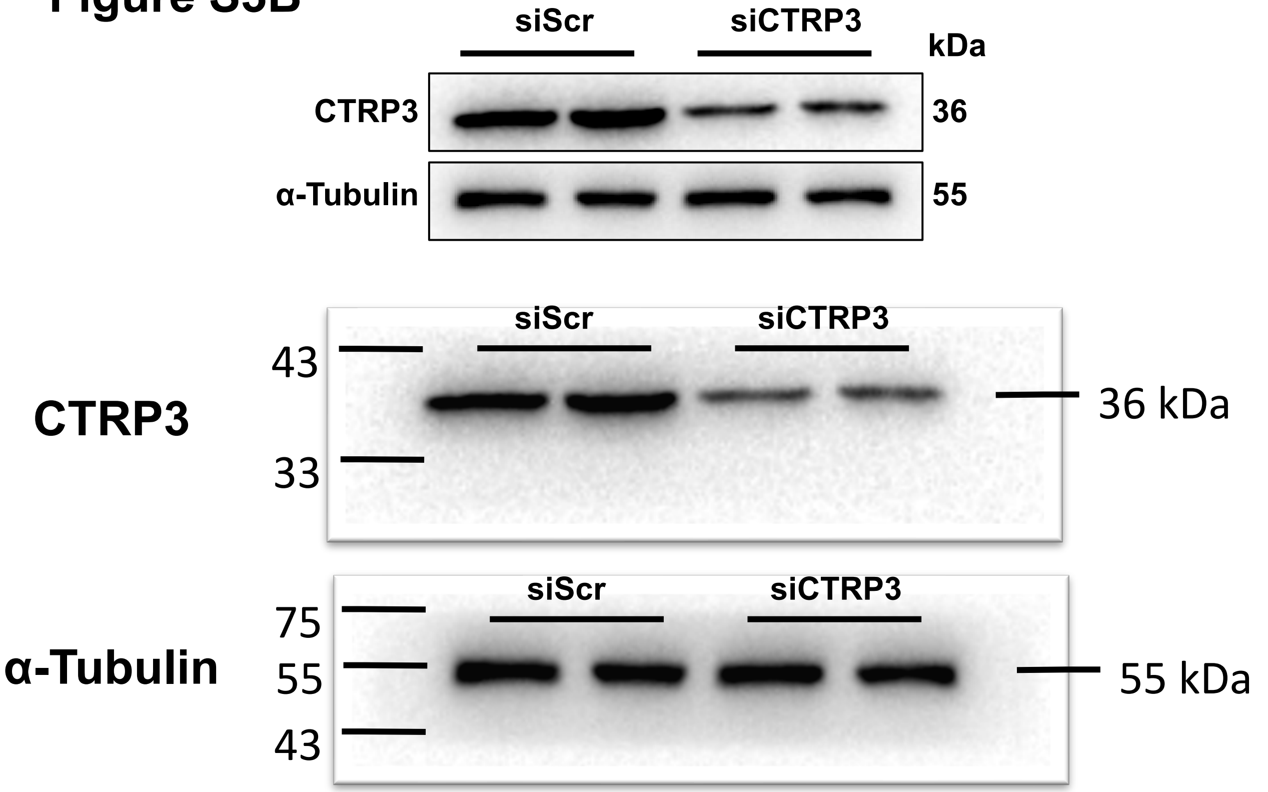

Supplement: Supplementary file 2 — Original Data File [file 41420_2024_1813_MOESM2_ESM.pdf]
